# Plasminogen Activator Inhibitor-1–Positive Platelet-Derived Extracellular Vesicles Predicts MACE and the Proinflammatory SMC Phenotype

**DOI:** 10.1016/j.jacbts.2022.05.002

**Published:** 2022-09-21

**Authors:** Richard G. Jung, Anne-Claire Duchez, Trevor Simard, Shan Dhaliwal, Taylor Gillmore, Pietro Di Santo, Alisha Labinaz, F. Daniel Ramirez, Adil Rasheed, Sabrina Robichaud, Mireille Ouimet, Spencer Short, Cole Clifford, Fengxia Xiao, Marie Lordkipanidzé, Dylan Burger, Suresh Gadde, Katey J. Rayner, Benjamin Hibbert

**Affiliations:** a CAPITAL Research Group, University of Ottawa Heart Institute, Ottawa, Ontario, Canada; b Vascular Biology and Experimental Medicine Laboratory, University of Ottawa Heart Institute, Ottawa, Ontario, Canada; c Department of Cellular and Molecular Medicine, Faculty of Medicine, University of Ottawa, Ottawa, Ontario, Canada; d Department of Biochemistry, Microbiology, and Immunology, Faculty of Medicine, University of Ottawa, Ottawa, Ontario, Canada; e Division of Cardiology, University of Ottawa Heart Institute, Ottawa, Ontario, Canada; f School of Epidemiology and Public Health, University of Ottawa, Ottawa, Ontario, Canada; g Faculty of Medicine, University of Ottawa, Ottawa, Ontario, Canada; h Kidney Research Centre, Department of Cellular and Molecular Medicine, Faculty of Medicine, University of Ottawa, Ottawa, Ontario, Canada; i Faculté de Pharmacie, Université de Montréal, Montréal, Québec, Canada; j Research Center, Montreal Heart Institute, Montréal, Québec, Canada

**Keywords:** biomarkers, extracellular vesicles, in-stent restenosis, percutaneous coronary intervention, plasminogen activator inhibitor-1, stent thrombosis, CAD, coronary artery disease, CMFDA, 5-chloromethylfluorescein diacetate, DAPT, dual antiplatelet therapy, DMSO, dimethyl sulfoxide, EV, extracellular vesicle, LRP1, low-density lipoprotein–related receptor-1, MACE, major adverse cardiac events, PAI, plasminogen activator inhibitor, PAI-1^+^ PEV, plasminogen activator inhibitor-1–positive platelet-derived extracellular vesicle, PCI, percutaneous coronary intervention, PEV, platelet-derived extracellular vesicle, *T-TAS*, Total Thrombus-formation Analysis System, VSMC, vascular smooth muscle cells

## Abstract

Patients with established coronary artery disease remain at elevated risk of major adverse cardiac events. The goal of this study was to evaluate the utility of plasminogen activator inhibitor-1–positive platelet-derived extracellular vesicles as a biomarker for major adverse cardiac events and to explore potential underlying mechanisms. Our study suggests these extracellular vesicles as a potential biomarker to identify and a therapeutic target to ameliorate neointimal formation of high-risk patients.

## Highlights

• This study shows the existence of PAI-1^+^ PEVs. Approximately 20% of plasma PAI-1 is composed of PAI-1^+^ PEVs.
• Elevated PAI-1^+^ PEV levels were predictive of 1-year major adverse cardiac events in both the discovery and the validation cohort, with larger effect sizes than other clinical biomarkers.
• High PAI-1^+^ PEV levels did not affect thrombogenicity.
• Increasing doses of PAI-1^+^ PEVs promoted the proinflammatory VSMC state by enhancing proliferation and migration. Inhibition of the PAI-1:low-density lipoprotein–related receptor-1 pathway dampened the proinflammatory VSMC changes.
• PAI-1^+^ PEV is a promising biomarker for major adverse cardiac events, and targeting the PAI-1^+^ PEV–VSMC interaction may offer a novel target to modulate cardiac events in patients with coronary artery disease.

## Visual Abstract

**Figure:**
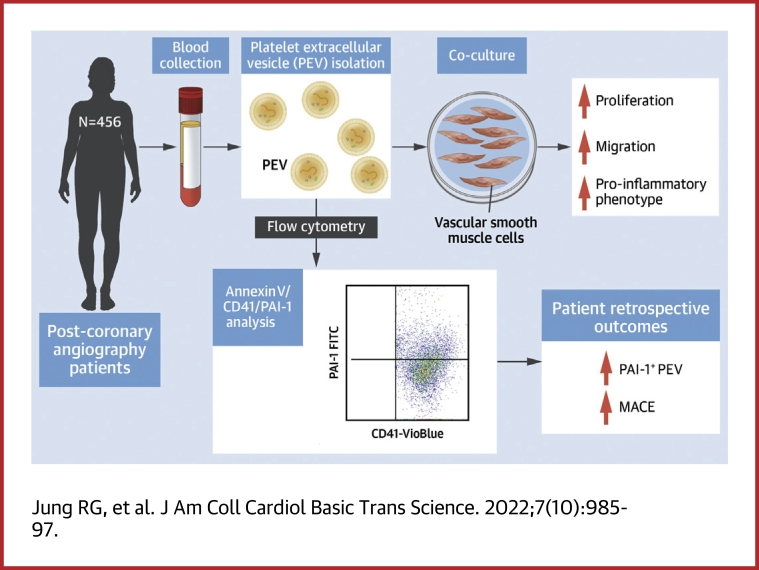

Obstructive coronary artery disease (CAD) is frequently treated with revascularization by percutaneous coronary intervention (PCI) or coronary artery bypass grafting in patients with refractory symptoms or acute coronary syndrome.^1^^,^^2^ Despite significant advances in PCI techniques and technologies since their inception, the stented coronary artery remains the highest risk coronary lesion, with annualized adverse event rates as high as 8% to 12% in the following year.^3^, ^4^, ^5^ Comparatively, the putative vulnerable plaque has an annualized event rate of 0.05%, making identification and treatment of patients after coronary stenting an unmet need. Two major complications resulting in target vessel failure are in-stent restenosis and stent thrombosis.^6^

Plasminogen activator inhibitor (PAI)-1 is primarily an antifibrinolytic protein that inhibits fibrin clot degradation by preventing the action of tissue-type plasminogen activator.^7^ Elevated levels of PAI-1 have been previously associated with type 2 diabetes, acute myocardial infarction, and unplanned revascularization.^8^, ^9^, ^10^ Of note, the majority of plasma PAI-1 is derived from platelets.^7^^,^^9^^,^^11^ Inhibition of PAI-1 has previously been shown to reduce vascular smooth muscle cell (VSMC) migration and prevent neointimal formation after vascular injury in mouse models.^12^^,^^13^ However, in humans, few studies have evaluated or linked PAI-1 levels to outcomes in patients with CAD.

Extracellular vesicles (EVs) are released from circulating blood and vascular cells at the time of vascular injury.^14^, ^15^, ^16^ In addition to small studies suggesting a role as a biomarker, EVs potentially modulate stent failure in 2 ways: 1) EVs are abundant in surface-exposed negatively charged phosphatidylserine, which catalyzes thrombosis^17^; and 2) EVs contain proinflammatory protein and genetic material that could potentially be transferred to recipient cells at sites of vascular injury to modulate a phenotypic response.^18^, ^19^, ^20^, ^21^, ^22^ To date, no data exist on the relationship between PAI-1 and EVs in humans despite platelets being a common source; there are also no data on if EVs play a pathologic role in clinical events in patients with CAD.

Accordingly, we hypothesized that platelet-derived EVs may represent a major source of pathologic PAI-1 (PAI-1–positive platelet-derived EVs [ie, PAI-1^+^ PEVs]). We also evaluated if these EVs were associated with major adverse cardiac events (MACE). Finally, we sought to evaluate the role of PAI-1^+^ PEVs in models of thrombus formation and in VSMC phenotypic switching for arterial remodeling post-revascularization.

## Methods

A detailed Methods section for all experiments are available in the Supplemental Methods section of the Supplemental Appendix. All the reagents and primers used for the study are available on Supplemental Tables 1 and 2. The evaluation of the utility of PAI-1^+^ PEVs as a biomarker was a retrospective analysis, whereas the in vitro evaluation of PAI-1^+^ PEVs was conducted prospectively. This study was approved by the Ottawa Health Science Network Research Ethics Board (#20160516-01H for in vitro experimentation and #20190224-01H for clinical follow-up). Written informed consent was obtained from all participants.

### Study population, data collection, and clinical outcomes

The University of Ottawa Heart Institute is a regional tertiary center serving >1.2 million people in the capital region of Canada, and all coronary catheterization procedures are registered in the CAPITAL (Cardiovascular and Percutaneous Clinical Trials) Revascularization Registry. This registry captures >1,200 clinical data points regarding patient and procedural factors.^23^ Patients undergoing revascularization in the evening were not included for workflow reasons as bloodwork was not captured in this population. Outpatient clinic or chart review was conducted at 1 year after the basal blood draw to evaluate the endpoints of MACE and unplanned revascularization. Clinicians who performed the clinical follow-up to determine adverse outcomes were blinded to PAI-1^+^ PEV levels. Clinical characteristics at 1 year were captured and recorded through the CAPITAL Revascularization Registry.

### Statistical analysis

Continuous variables are reported as mean ± SD or median with 25th and 75th percentiles (quartile 1-quartile 3) as appropriate. Normality was assessed by using the Shapiro-Wilks test along with visual inspection of Q-Q plots and subsequently compared by using Student’s *t*-test or Mann-Whitney *U* test with parametric and nonparametric data, respectively. Ad hoc multiple pairwise comparisons vs either dimethyl sulfoxide (DMSO) or EV controls were evaluated by using Dunnett's test. Categorical variables are reported as counts and percentages. Categorical variables were compared by using the chi-square test or Fisher’s exact test.

All patients included in the study completed their 1-year follow-up. Receiver-operating characteristic curves were generated, and the Youden index was identified to ascertain the optimal cutoff value to simultaneously maximize sensitivity and specificity of PAI-1^+^ PEVs in the discovery cohort and validated in the validation cohort.^24^ Kaplan-Meier curves were generated to evaluate time-to-event distributions and compared by using log-rank tests. HRs and corresponding 95% CIs were obtained from Cox proportional hazards models after adjustment by clinical characteristics (age, type 2 diabetes, and acute coronary syndrome) determined a priori based on clinical judgment of variables influencing MACE.

All flow cytometry data were analyzed by using FlowJo version 10 (Becton, Dickinson & Company), and all statistical analyses were performed by using SAS version 9.4 (SAS Institute, Inc). Receiver-operating characteristic curves and reclassification were performed by using the %ROCPLUS macro. All figures were created by using GraphPad Prism version 8 (GraphPad Software). Values of *P* < 0.05 were considered statistically significant.

## Results

### PAI-1^+^ PEVs in human circulation

**Figure 1:**
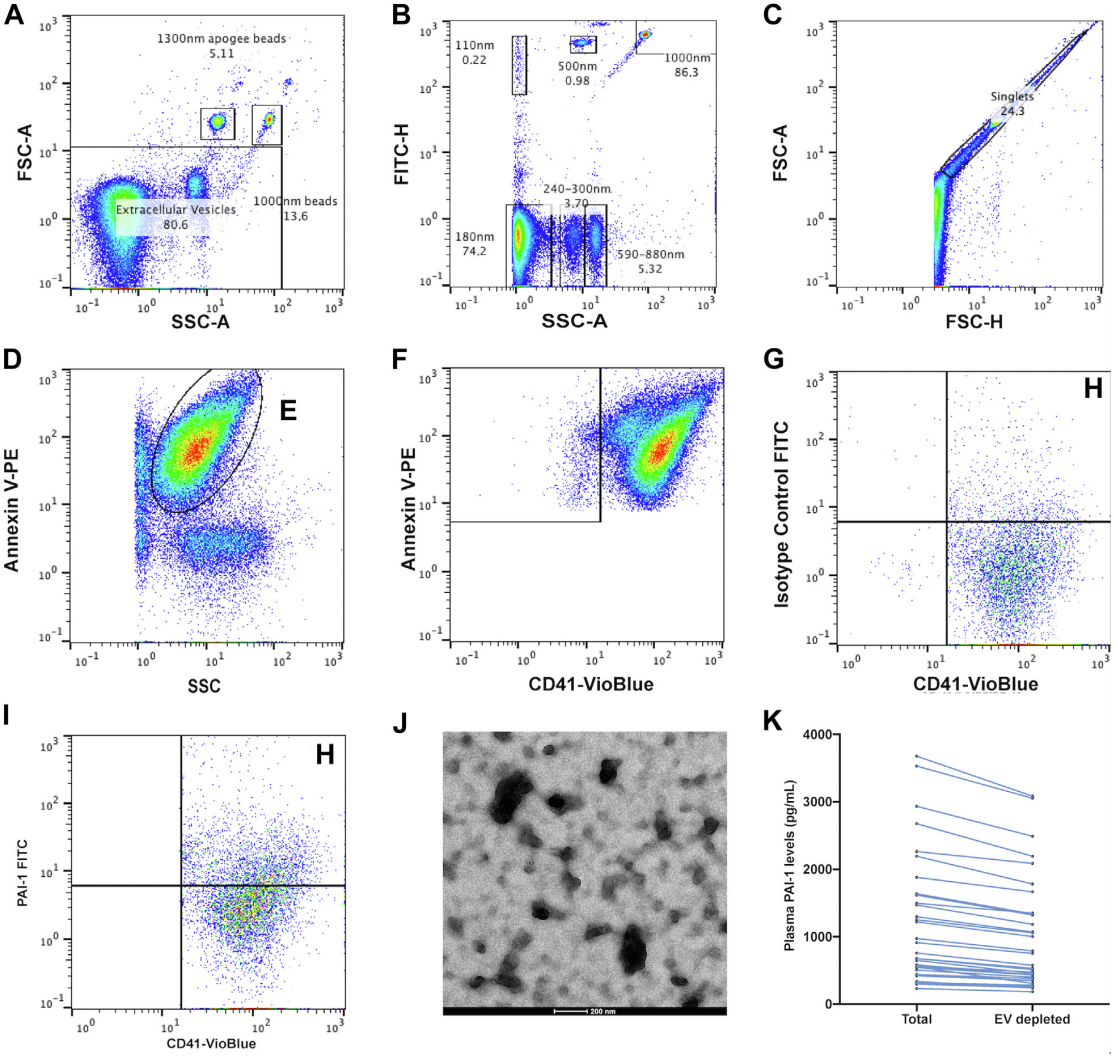
Flow Cytometry Gating Profile to Identify PAI-1^+^ PEV **(A, B)** Forward scatter (FSC) and side scatter (SSC) gate to identify upper limit using Apogee Beads. Representative Fluoresbrite YG beads (Polysciences, Inc) are labeled 1,000-nm beads. **(C)** Singlet discrimination using FSC–area (FSC-A) and FSC–height (FSC-H). **(D)** Annexin V-PE–positive gate (gate labeled **E**) to isolate extracellular vesicles. **(F)** From Annexin V^+^ gate, platelet extracellular vesicles (CD41^+^) were identified. **(G, I)** Gating profile of plasminogen activator inhibitor-1–positive platelet-derived extracellular vesicle (PAI-1^+^ PEV) identified H-gate (gate labeled **H**) using isotype control fluorescein isothiocyanate (FITC). **(J)** PAI-1^+^ PEV complex captured by electron microscopy after immunogold staining of PAI-1. **(K)** Plasma PAI-1 levels measured at baseline and EV-depleted samples. All gates were drawn based on fluorescence minus one control unless otherwise specified. FITC-H = fluorescein isothiocyanate-H; SSC-A = side scatter–area; EV = extracellular vesicle.

After isolation of EVs from human plasma in patients with CAD spun at 20,000 *g*, PAI-1^+^ PEVs (defined as Annexin V^+^/CD41^+^/PAI-1^+^ large EVs) were measured by using flow cytometry after generating a size gate using standardized Apogee beads (Apogee Flow Systems). Gates for Annexin V, CD41, and PAI-1 were based on the use of isotype and fluorescence minus one control where appropriate (Figures 1A to 1I). Localization of PAI-1 on the surface of PEV was further validated by using electron microscopy by labeling with PAI-1 antibody and appropriate gold-linked immunoglobulin G secondary antibody (Figure 1J). To determine the proportion of PAI-1 associated with EVs in human plasma, plasma was EV-depleted (Figure 1K shows the relative decrease in PAI-1 levels), which demonstrates that PAI-1^+^ PEV contributes to 19.1% ± 6.3% of PAI-1 levels in plasma.

### Plasma PAI-1^+^ PEV does not affect thrombus formation

**Figure 2:**
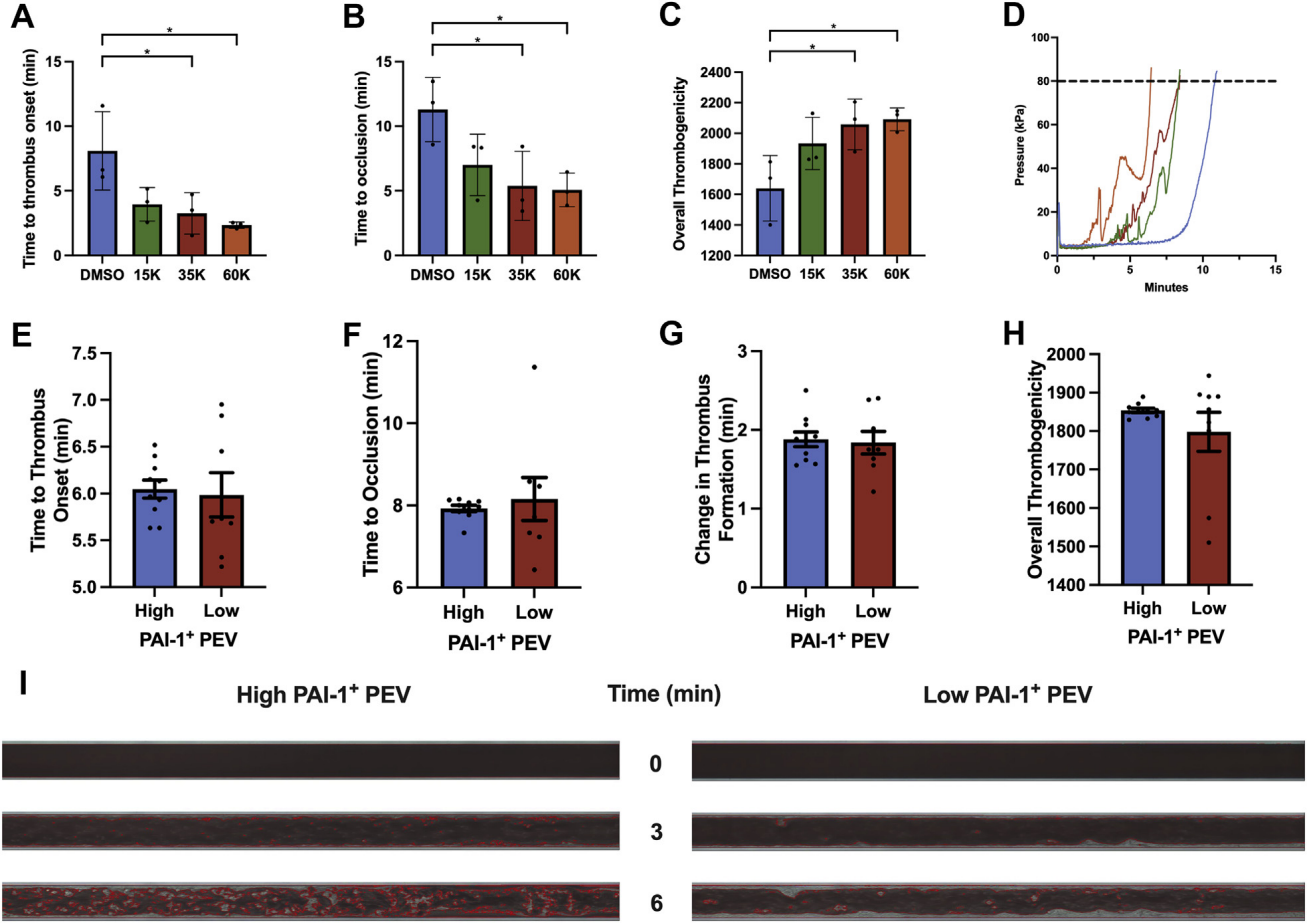
Impact of PEV and PAI-1^+^ PEV on Thrombus Formation Using T-TAS **(A)** Time to onset of thrombus (time to reach 10 kPa) decreased with increasing PEV (8.1 ± 3.0 minutes vs 4.0 ± 1.3 minutes vs 3.3 ± 1.6 minutes vs 2.3 ± 0.2 minutes, for dimethyl sulfoxide [DMSO], 15,000 [15K], 35,000 [35K], and 60,000 [60K] PEV/μL, respectively; n = 3). **(B)** Time to thrombus occlusion (time to reach 80 kPa) decreased with increasing PEV (11.3 ± 2.5 minutes vs 7.0 ± 2.4 minutes vs 5.4 ± 2.7 minutes vs 5.1 ± 1.3 minutes for DMSO, 1.5 × 10^4^, 3.5 × 10^4^, and 6.0 × 10^4^ PEV/μL; n = 3). **(C)** Overall thrombogenicity increased with increasing concentrations of PEV (1,640 ± 214 vs 1,934 ± 171 vs 2,059 ± 166 vs 2,092 ± 75 for DMSO, 1.5 × 10^4^, 3.5 × 10^4^, and 6.0 × 10^4^ PEV/μL; n = 3). **(D)** Average pressure vs time curve for DMSO **(blue)**, 1.5 × 10^4^**(green)**, 3.5 × 10^4^**(red)**, and 6.0 × 10^4^ PEV/μL **(orange)**. **(E)** Time to onset of thrombus did not differ between PAI-1^+^ PEV fractions (6.0 ± 0.3 minutes vs 6.0 ± 0.7 minutes for high vs low; n = 10 and n = 8; *P =* 0.80). **(F)** Time to thrombus occlusion was not different between PAI-1^+^ PEV fractions (7.9 ± 0.2 minutes vs 8.2 ± 1.5 minutes for high vs low; n = 10 and n = 8; *P =* 0.63). **(G)** No differences in rate of thrombus formation was observed between PAI-1^+^ PEV fractions (1.9 ± 0.3 minutes vs 1.8 ± 0.4 minutes for high vs low; n = 10 and n = 8; *P =* 0.80). **(H)** Overall thrombogenicity was not different between PAI-1^+^ PEV fractions (1,854.0 ± 18.0 area under the curve [AUC] vs 1,798.0 ± 151.8 AUC for high vs low; n = 10 and n = 8; *P =* 0.27). **(I)** Corresponding microscopic images of thrombus formation of low vs high PAI-1^+^ PEV fractions under flow conditions in the AR-chip. ∗*P <* 0.05. Abbreviations as in Figure 1.

We hypothesized that PAI-1^+^ PEVs would mediate thrombosis as both PAI-1 and EVs independently propagate thrombus formation. Using the available clinical data, no relationship was observed between PAI-1^+^ PEV levels in CAD patients with or without antiplatelet therapy, nor was there an association between antiplatelets and MACE (Supplemental Figure 1). We next evaluated the effect of PEVs on thrombogenicity using the Total Thrombus-formation Analysis System (*T-TAS*).^25^ Increasing concentrations of PEVs (1.5-6.0 × 10^4^ PEV/μL) were added to healthy volunteer blood and were associated with rapid thrombus initiation and capillary occlusion along with increased thrombogenicity (n = 3) (Figures 2A to 2D). To determine the effect of PAI-1^+^ PEV on thrombus formation, we isolated plasma EVs from high vs low proportions of PAI-1^+^ PEV patients and incubated healthy volunteer blood with a standardized amount of PEVs (3.5 × 10^4^ PEV/μL). The incubated blood was processed on the T-TAS, where no differences were observed in time to onset of thrombus formation between high vs low proportions of PAI-1^+^ PEV (6.0 ± 0.3 minutes vs 6.0 ± 0.7 minutes; *P =* 0.80; n = 10 and n = 8, respectively) (Figure 2E) or time to occlusion (7.9 ± 0.2 minutes vs 8.2 ± 1.5 minutes; *P =* 0.63; n = 10 and n = 8) (Figure 2F). Moreover, the rate of thrombus formation, calculated as a difference from time of onset to occlusion, did not differ between the 2 groups (1.9 ± 0.3 minutes vs 1.8 ± 0.4 minutes; *P =* 0.80; n = 10 and n = 8) (Figure 2G). Overall, thrombogenicity remained similar between the 2 groups (1,854.0 ± 18.0 area under the flow pressure curve vs 1,798.0 ± 151.8 area under the flow pressure curve; *P =* 0.27; n = 10 and n = 8) (Figure 2H). Corresponding microscopic images of thrombus formation under AR-chip (Fujimori Kogyo Co., Yokohama, Japan) flow conditions are presented in Figure 2I. Although increasing concentrations of PEV affected thrombus formation, different proportions of PAI-1^+^ PEV do not seem to affect thrombogenicity.

### PAI-1^+^ PEVs modulate vsmc phenotypic switching

**Figure 3:**
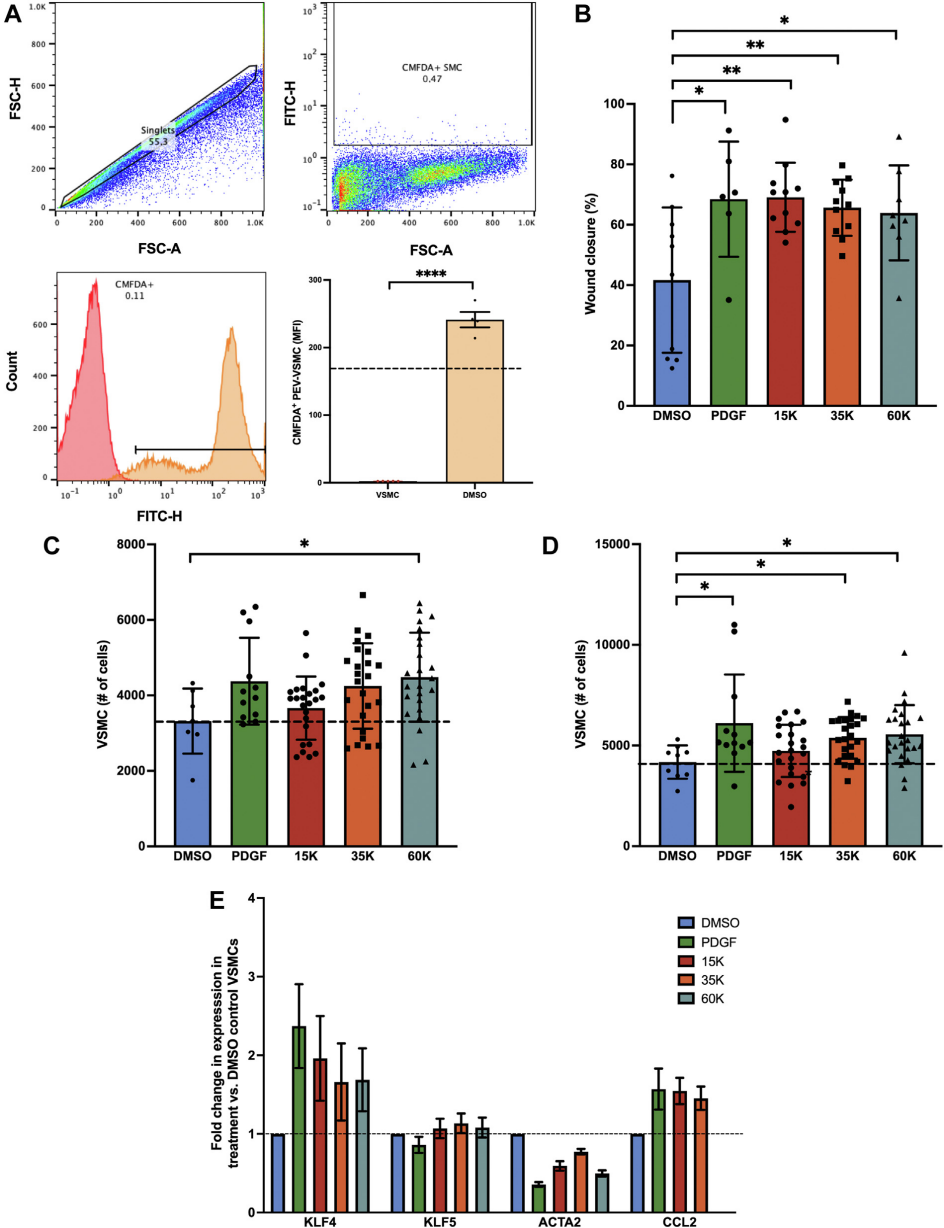
PEVs Modulate VSMC Phenotypic Switching **(A)** 5-Chloromethylfluorescein diacetate (CMFDA)^+^ PEV adhesion is observed in smooth muscle cells (170.7 ± 98.3 vs 2.5 ±0.02; *P =* 0.005). **(B)** Incubation of vascular smooth muscle cells (VSMCs) with PEVs enhanced migration (wound closure) 1.7-fold compared with DMSO control at 24 hours (41.7% ± 24.1% [n = 10], 68.5% ± 19.1% [n = 6], 69.1% ± 11.5% [n = 11], 65.6% ± 9.3% [n = 11], and 63.9% ± 15.7% [n = 8], for DMSO, platelet-derived growth factor [PDGF], 1.5 × 10^4^, 3.5 × 10^4^, and 6.0 × 10^4^ PEV/μL, respectively). **(C)** CellTrace Violet^+^ VSMC proliferation in the presence of increasing concentrations of PEVs at 24 hours. Only 60,000 PEV/μL promoted proliferation compared with DMSO control (4,481.0 ± 1,179.3 VSMCs vs 3,319.3 ± 863.3 VSMCs, 6.0 × 10^4^ PEV/μL [n = 24] and DMSO [n = 7]; *P =* 0.02). **(D)** CellTrace Violet^+^ VSMC proliferation in the presence of increasing concentrations of PEVs at 48 hours. Both 3.5 × 10^4^ and 6.0 × 10^4^ PEVs/μL promoted proliferation compared with DMSO control (5,473.1 ± 945.4 VSMCs vs 5,658.5 ± 1,396.5 VSMCs vs 4,357.9 ± 673.5 VSMCs, for 35,000 PEVs/μL (n = 24), 6.0 × 10^4^ PEVs/μL (n = 24), and DMSO (n = 10); *P =* 0.004). **(E)** VSMC co-cultured with PEVs exhibited changes in the VSMC differentiation markers KLF4, ACTA2, and CCL2. 15K, 35K, and 60K represent 1.5 × 10^4^, 3.5 × 10^4^, and 6.0 × 10^4^ PEV/μL. ∗*P <* 0.05, ∗∗*P <* 0.01. Abbreviations as in Figures 1 and 2.

Next, we studied the effect of PAI-1^+^ PEVs on VSMC phenotypic switching by using a combination of functional assays and changes in VSMC cellular programming. First, PEV adhesion (1.0 × 10^8^/mL) with VSMC incubated in suspension (5.0 × 10^5^/mL) was assessed by flow cytometry after staining PEVs with CellTracker 5-chloromethylfluorescein diacetate (CMFDA). The interaction between PEV and VSMC was quantified as an increase in median fluorescence intensity relative to the unstained VSMC fraction (170.7 ± 98.3 vs 2.5 ± 0.02; *P =* 0.005; n = 5) (Figure 3A). Similarly, co-incubation of CMFDA^+^ PEVs with VSMCs displays PEV binding in a dose- and time-dependent fashion to VSMCs (Supplemental Figure 2).

We next evaluated the effect of PAI-1^+^ PEVs on VSMC migration through an in vitro scratch assay in the presence of increasing concentrations of PEVs (Supplemental Figure 3). Incubation of SMC with PEVs enhanced wound closure 1.7-fold compared with DMSO control at 24 hours (41.7% ± 24.1% [n = 10], 68.5% ± 19.1% [n = 6], 69.1% ± 11.5% [n = 11], 65.6% ± 9.3% [n = 11], and 63.9% ± 15.7%, [n = 8] for DMSO, platelet-derived growth factor, 1.5 × 10^4^ PEV/μL, 3.5 × 10^4^ PEV/μL, and 6.0 × 10^4^ PEV/μL, respectively) (Figure 3B, Supplemental Figure 3). Proliferation was evaluated by using CellTrace Violet–stained VSMCs at baseline and evaluated by using flow cytometry in the presence of increasing concentrations of PEVs. After 24 hours of incubation, only 6.0 × 10^4^ PEV/μL promoted proliferation compared with DMSO control (4,481.0 ± 1,179.3 VSMCs vs 3,319.3 ± 863.3 VSMCs, 6.0 × 10^4^ PEV/μL (n = 24) and DMSO (n = 7); *P =* 0.02) (Figure 3C). After 48 hours of incubation, 3.5 × 10^4^ PEVs/μL promoted proliferation compared with DMSO control (5,473.1 ± 945.4 VSMCs vs 5,658.5 ± 1,396.5 VSMCs vs 4,357.9 ± 673.5 VSMCs, for 3.5 × 10^4^ PEVs/μL [n = 24], 6.0 × 10^4^ PEVs/μL [n = 24], and DMSO [n = 10]; *P =* 0.004) (Figure 3D). Finally, VSMC co-cultured with PEVs exhibited changes in the VSMC trans-differentiation markers KLF4, ACTA2, and CCL2 (Figure 3E). Similarly, PEVs enhanced procalcification osteogenic changes of vascular SMCs with an increase in bone morphogenetic protein 2 expression (Supplemental Figure 4).

### PAI-1^+^ PEV is elevated in mace

The baseline characteristics of 456 patients who had a 1-year clinical follow-up with enumerated PAI-1^+^ PEVs are presented in Table 1. Briefly, the mean age of the cohort was 66.7 ± 11.3 years, and 124 patients (27.2%) were female. Vascular risk factors include hypertension (66.2%), dyslipidemia (60.8%), diabetes (32.2%), active smoking (17.3%), and family history of CAD (14.3%). Importantly, patients had mixed indications for angiography, including both stable CAD (36.2%) and acute coronary syndrome (44.5%), and 270 patients (59.2%) underwent revascularization in the form of PCI or coronary artery bypass grafting. Finally, 357 patients (78.3%) had ≥1 vessel-burden with significant CAD.

**Table 1:** Patient Baseline Characteristics

	Total (N = 456)	Quartile 1 (n = 114)	Quartile 2 (n = 117)	Quartile 3 (n = 112)	Quartile 4 (n = 113)	*P* Value
Age, y	66.7 ± 11.3	67.4 ± 10.9	65.4 ± 11.4	68.2 ± 11.3	65.8 ± 11.7	0.21
Female	124 (27.2)	31 (27.2)	29 (24.8)	34 (30.4)	30 (26.6)	0.82
Hypertension	302 (66.2)	76 (66.7)	76 (65.0)	80 (71.4)	70 (62.0)	0.50
Dyslipidemia	277 (60.8)	74 (64.9)	67 (57.3)	69 (61.6)	67 (59.3)	0.67
Diabetes	147 (32.2)	35 (31.0)	33 (28.2)	45 (40.5)	34 (30.9)	0.21
Smoking						0.35
Never	265 (58.1)	69 (60.5)	74 (63.3)	62 (55.4)	60 (53.1)	
Remote (quit >1 mo ago)	112 (24.6)	29 (25.4)	23 (19.7)	33 (29.5)	27 (23.9)	
Active	79 (17.3)	16 (14.0)	20 (17.1)	17 (15.2)	26 (23.0)	
Family history of CAD	65 (14.3)	21 (18.4)	15 (12.8)	14 (12.5)	15 (13.3)	0.53
Atrial fibrillation	48 (10.5)	17 (14.9)	11 (9.4)	12 (10.7)	8 (7.1)	0.27
Indications for angiography						
Acute coronary syndrome	203 (44.5)	40 (35.1)	53 (45.3)	49 (43.8)	61 (54.0)	0.041
Staged PCI	42 (9.2)	10 (8.8)	12 (10.3)	12 (10.7)	8 (7.1)	0.78
Stable CAD	165 (36.2)	53 (46.5)	36 (30.8)	41 (36.6)	35 (31.0)	0.045
Shock	1 (0.2)	1 (0.9)	0	0	0	0.39
Medical history						
PCI	118 (25.9)	32 (28.1)	24 (20.5)	35 (31.3)	27 (23.9)	0.27
Myocardial infarction	85 (18.6)	25 (21.9)	20 (17.1)	19 (17.0)	21 (18.6)	0.75
CABG	23 (5.0)	6 (5.3)	2 (1.7)	11 (9.8)	4 (3.5)	0.035
Peripheral artery disease	36 (7.9)	6 (5.3)	10 (8.6)	12 (10.7)	8 (7.1)	0.48
Cerebrovascular accident	27 (5.9)	5 (4.4)	6 (5.1)	10 (8.9)	6 (5.3)	0.47
Bleed	8 (1.8)	2 (1.8)	1 (0.9)	3 (2.7)	2 (1.8)	0.78
Heart failure	23 (5.0)	5 (4.4)	4 (3.4)	8 (7.1)	6 (5.3)	0.62
Medications						
Aspirin	415 (91.0)	101 (88.6)	107 (91.5)	103 (92.0)	104 (92.0)	0.77
P2Y_12_ inhibitor	410 (89.9)	102 (89.5)	102 (87.2)	105 (93.8)	101 (89.4)	0.42
ACE inhibitor/ARB	238 (52.2)	61 (53.5)	62 (53.0)	53 (47.3)	62 (54.9)	0.68
Beta-blocker	268 (58.8)	71 (62.3)	66 (56.4)	60 (53.6)	71 (62.8)	0.41
Calcium channel blocker	63 (13.8)	19 (16.7)	15 (12.8)	17 (15.2)	12 (10.6)	0.57
Statin	372 (81.6)	92 (80.7)	93 (79.5)	95 (84.8)	92 (81.4)	0.76
Proton pump inhibitor	71 (15.6)	22 (19.3)	15 (12.8)	16 (14.3)	18 (15.9)	0.57
No. of vessels with obstructive (≥50%) CAD						0.83
0	99 (21.7)	23 (20.2)	25 (21.4)	26 (23.2)	25 (22.1)	
1	193 (42.3)	48 (42.1)	56 (47.9)	45 (40.2)	44 (38.9)	
≥2	164 (36.0)	43 (37.7)	36 (30.8)	41 (36.6)	44 (38.9)	
Revascularized (PCI + CABG)	270 (59.2)	69 (60.5)	79 (67.5)	64 (57.1)	58 (51.3)	0.09

Values are mean ± SD or n (%), unless otherwise indicated.

ACE/ARB = angiotensin-converting enzyme/angiotensin II receptor blocker; CABG = coronary artery bypass grafting; CAD = coronary artery disease; PCI = percutaneous coronary intervention.

During a median follow-up period of 12.7 months (quartile 1-quartile 3: 10.6-15.2 months), 76 patients (16.7%) in our cohort experienced MACE, with 36 deaths, 7 myocardial infarctions, 28 unplanned revascularizations, and 18 cerebrovascular accidents. When stratified according to MACE, differences in rates of previous PCI and myocardial infarction were observed (Supplemental Table 3). Patients who had MACE had significantly elevated median PAI-1^+^ PEV levels (13,786.6 [quartile 1-quartile 3: 6,711.7-26,942.1] PAI-1^+^ PEV/μL vs 8,344.9 [quartile 1-quartile 3: 3,777.2-33,499.7] PAI-1^+^ PEV/μL [n = 76 and n = 380], respectively; *P =* 0.047) (Supplemental Figure 5A) and elevated median PEV levels (107,463.0 [quartile 1-quartile 3: 58,416.1-202,987.8] PEV/μL vs 71,675.6 [quartile 1-quartile 3: 31,184.5-201,313.3] PEV/μL [n = 76 and n = 380]; *P =* 0.04) (Supplemental Figure 5B). No differences were observed in plasma PAI-1 levels and (PAI-1^+^ PEV)/PEV fraction between event and no-event (Supplemental Figures 5C and 5D).

### Utility of PAI-1^+^ PEV as a biomarker of mace: discovery and validation cohort

**Figure 4:**
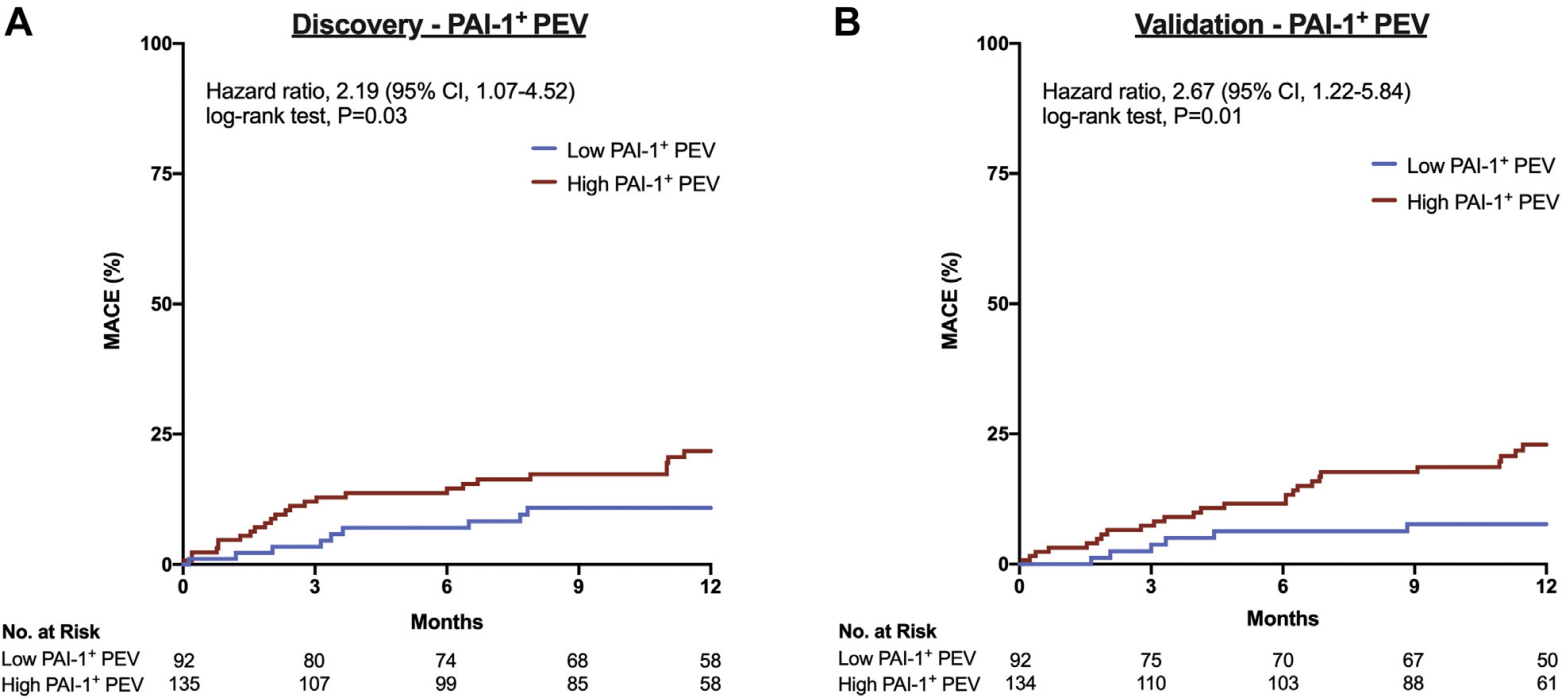
Utility of PAI-1^+^ PEV Complex for Prediction of MACE **(A)** In the discovery cohort patients with cumulative incidence of major adverse cardiac events (MACE) within 1-year follow-up, high PAI-1^+^ PEV levels were predictive of MACE (HR: 2.19; 95% CI: 1.07-4.52; *P =* 0.03). **(B)** In the validation cohort, patients with cumulative incidence of MACE within 1-year follow-up, high PAI-1^+^ PEV levels remained predictive of MACE (HR: 2.67; 95% CI: 1.22-5.84; *P =* 0.01). Kaplan-Meier curves were generated and compared by using log-rank tests, and HRs were evaluated by using the Cox proportional hazards model. Abbreviations as in Figures 1 and 2.

In an all-comer population, we sought to evaluate whether PAI-1^+^ PEV levels were predictive of MACE, and the cohort was equally divided into a discovery and a validation cohort (Supplemental Table 4). The optimal cutoff point (Youden’s index) identified in the discovery cohort for prediction of MACE using PAI-1^+^ PEV was ≥ 6,321.9/μL, with a sensitivity of 0.79 (95% CI: 0.63-0.90) and a specificity of 0.43 (95% CI: 0.36-0.51) along with a crude MACE rate of 262.3 vs 103.0 events per 1,000 person-years for high vs low PAI-1^+^ PEV levels, respectively. High PAI-1^+^ PEV levels were associated with MACE with an HR of 2.43 (95% CI: 1.43-4.13; *P =* 0.001) (Table 2, Model 1). When adjusted for clinical variables associated with MACE (age, type 2 diabetes, sex, and acute coronary syndrome), high PAI-1^+^ PEV levels remained associated with MACE, with an HR of 2.49 (95% CI: 1.46-4.25; *P =* 0.001), and PAI-1^+^ PEV was the strongest predictor of MACE compared with the best known clinical variables (Table 2, Model 2). In the discovery cohort, high PAI-1^+^ PEV levels were associated with MACE, with an HR of 2.19 (95% CI: 1.07-4.52; log-rank test; *P =* 0.03) (Figure 4A). In the validation cohort, high PAI-1^+^ PEV levels remained predictive of MACE, with an HR of 2.67 (95% CI: 1.22-5.84; log-rank test; *P =* 0.01) (Figure 4B).

**Table 2:** MACE Predicted According to PAI-1^+^ PEV Levels

	PAI-1^+^ PEV	
	HR (95% CI)	*P* Value
Model 1: unadjusted PAI-1^+^ PEV levels		
PAI-1^+^ PEV cutoff at Youden's index	2.43 (1.43-4.13)	0.001
Model 2: adjusted by age (y), acute coronary syndrome, sex, and type 2 diabetes		
PAI-1^+^ PEV cutoff at Youden’s index	2.49 (1.46-4.25)	0.001
Age (y)	1.06 (1.04-1.08)	<0.0001
Acute coronary syndrome	1.19 (0.75-1.89)	0.46
Sex	1.07 (0.65-1.75)	0.80
Type 2 diabetes	1.75 (1.11-2.76)	0.017

MACE = major adverse cardiac events; PAI-1^+^ PEV = plasminogen activator inhibitor-1–positive platelet-derived extracellular vesicle.

### Inhibition of PAI-1 attenuates smc phenotypic switch but not thrombus formation

**Figure 5:**
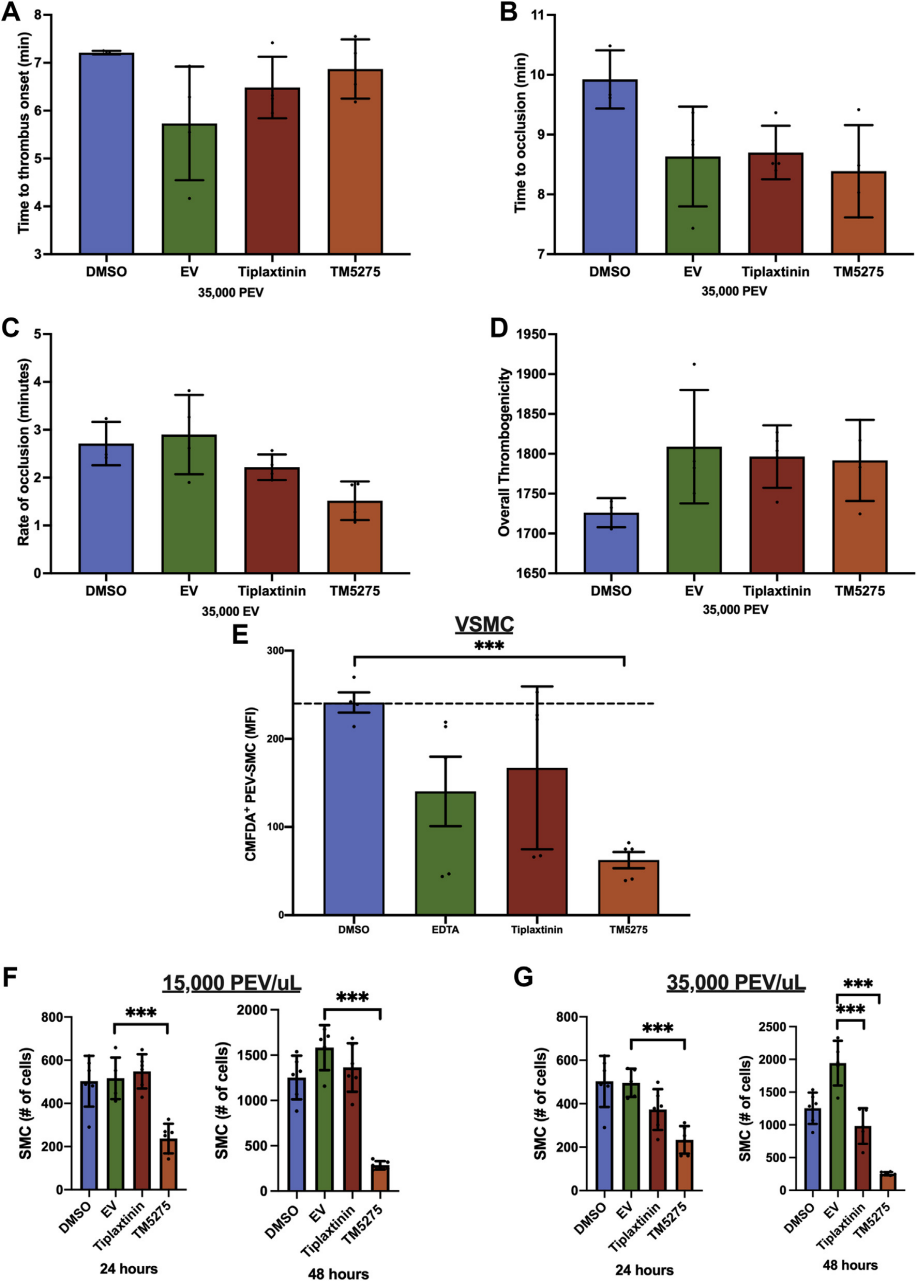
Inhibition of PAI-1 Does Not Affect Thrombus Formation but Attenuates the Proinflammatory VSMC Phenotype **(A)** No difference in time to onset of thrombus was observed in the presence of PAI-1 inhibitors (5.7 ± 1.2 minutes vs 6.5 ± 0.6 minutes vs 6.9 ± 0.6 minutes for EV, 25 μM tiplaxtinin, and 0.1 μM TM5275, respectively; n = 4; *P =* 0.80). **(B)** Time to thrombus occlusion was not different in the presence of PAI-1 inhibitors (8.6 ± 0.8 minutes vs 8.7 ± 0.4 minutes vs 8.4 ± 0.8 minutes for EV, 25 μM tiplaxtinin, and 0.1 μM TM5275; n = 4; *P =* 0.63). **(C)** No difference in rate of thrombus formation was observed in the presence of PAI-1 inhibitors (2.9 ± 0.8 minutes vs 2.2 ± 0.3 minutes vs 1.5 ± 0.4 minutes for EV, 25 μM tiplaxtinin, and 0.1 μM TM5275; n = 4; *P =* 0.80). **(D)** Overall thrombogenicity was not different in the presence of PAI-1 inhibitors (1,809 ± 71 AUC vs 1,797 ± 39 AUC vs 1,792 ± 51 AUC for EV, 25 μM tiplaxtinin, and 0.1 μM TM5275; n = 4; *P =* 0.27). **(E)** CMFDA^+^ PEV and VSMC interaction was attenuated in the presence of 0.1 μM TM5275 (62.5 ± 20.6 mean fluorescence intensity [MFI] vs 241.3 ± 22.9 MFI; n = 5; *P <* 0.001) but not with 25 μM tiplaxtinin (167.1 ± 92.4 vs 241.3 ± 22.9; n = 5; *P =* 0.25). **(F)** VSMC proliferation was inhibited in the presence of TM5275 after 24- and 48-hour incubation of 15,000 PEV/μL. **(G)** VSMC proliferation was inhibited in the presence of TM5275 after 24- and 48-hour incubation of 3.5 × 10^4^ PEV/μL. ∗*P <* 0.05, ∗∗*P <* 0.01, ∗∗∗*P <* 0.001. Abbreviations as in Figures 1, 2, and 3.

Finally, the functional effect of PAI-1 on PAI-1^+^ PEV was evaluated by using 2 known PAI-1 inhibitors, TM5275 (inhibits PAI-1 and low-density lipoprotein–related receptor-1 [LRP1] interaction) and tiplaxtinin (inhibits PAI-1 by binding to vitronectin-binding site), after validating PAI-1 activity on the surface of PAI-1^+^ PEVs (Supplemental Figure 6). Neither of the 2 inhibitors affected thrombus formation in T-TAS; there was no change in onset of thrombus formation, occlusion, rate of thrombus formation, or overall thrombogenicity (Figures 5A to 5D). The addition of TM5275 reduced CMFDA^+^ PEV and VSMC interaction by 3.7-fold (62.5 ± 20.6 mean fluorescence intensity vs 241.3 ± 22.9 mean fluorescence intensity; *P <* 0.001) (Figure 5E), whereas tiplaxtinin had no effect on PEV-VSMC interaction (167.1 ± 92.4 vs 241.3 ± 22.9; *P =* 0.25). TM5275 significantly reduced VSMC proliferation regardless of the PEV dosage (1.5 × 10^4^ or 3.5 × 10^4^ PEV/μL) at both the 24- and 48-hour time points (Figures 5G and 5H).

## Discussion

Patients with CAD continue to experience adverse events despite guideline-directed medical therapy and revascularization. Identification of novel markers to risk-stratify patients and/or to offer new therapeutic interventions remains a priority. Given the potential of EVs to behave as a biomarker and due to the common origin of PAI-1 and EV from platelets, our study sought to evaluate whether PAI-1^+^ PEV predicts MACE and affects thrombus formation and the proinflammatory VSMC phenotype. Our study presents 5 novel findings regarding PAI-1^+^ PEV: 1) the presence of a PAI-1^+^ PEV complex in human plasma; 2) PAI-1^+^ PEVs in standardized PEV fraction have no effect on thrombus formation; 3) PEV promotes the proinflammatory and pro-osteogenic VSMC phenotype; 4) inhibition of PAI-1^+^ PEVs through TM5275 attenuates VSMC phenotypic switching but has no effect on thrombus formation; and 5) high PAI-1^+^ PEV levels were predictive of MACE in both the discovery and the validation cohort. Overall, PAI-1^+^ PEVs act as a promising biomarker and offer a novel target to modulate vessel biology in patients with CAD.

We hypothesized that circulating plasma PAI-1 was largely EV derived and would modulate event rates in patients with CAD by increasing thrombotic risk. We found that 19.1% of circulating PAI-1 is in PAI-1^+^ PEVs, a newly identified subgroup of circulating PEVs. In our in vitro models, PAI-1^+^ PEVs did not increase thrombogenicity compared with PEVs alone. Interestingly, PEVs were shown to interact with VSMCs, subsequently increasing phenotypic switching, migration, and synthetic function. Inhibition models confirmed that VSMCs sequestered PEVs through a PAI-1– and LRP1-dependent interaction resulting in VSMC changes. Finally, high levels of circulating PAI-1^+^ PEVs were strongly associated with adverse clinical outcomes in our cohort, suggesting a strong potential as a biomarker of adverse events in high-risk patients.

PAI-1 mainly functions as an antiproteolytic protein but also appears to serve a ligand role affecting migration, proliferation, and apoptosis at sites of vascular injury.^26^, ^27^, ^28^, ^29^ The current study found that increasing concentrations of PAI-1^+^ PEV steers the pathologic changes observed in proinflammatory VSMCs to drive neointimal formation. Specifically, PAI-1 was previously shown to interact with LRP1 to stimulate the JAK/STAT-1 signaling cascade, driving VSMC migration.^27^ Indeed, PAI-1 inhibition with TM5275, an inhibitor of PAI-1 and LRP1 interaction, greatly attenuated the PEV–VSMC interaction and VSMC proliferation in our model, whereas tiplaxtinin had no effect. This observation is crucial, as TM5275 inhibits PAI-1 interaction with LRP1, whereas tiplaxtinin inhibits PAI-1 by binding to its vitronectin-binding site.^30^ Although we do not understand how PAI-1 remains bound on the surface of PEVs, we hypothesize that a complex formed between vitronectin (and its associated integrin receptor) with PAI-1 protects it from tiplaxtinin to serve as a signaling ligand of the PAI-1^+^ PEV complex.^31^ Indeed, the effect of PAI-1^+^ PEV modulation on the proinflammatory VSMC phenotype was attenuated with TM5275 and is a promising therapeutic target to reduce neointimal formation.

Finally, our results show that elevated levels of PAI-1^+^ PEV were strongly predictive of MACE out to 1 year. This study adds to the existing literature in that EVs have strong utility to predict cardiovascular events and are promising targets as they are the major hub linking inflammation, vascular injury, coagulation, and cellular reprogramming responsible for both acute and chronic complications.^20^^,^^32^, ^33^, ^34^ In the current study, PAI-1^+^ PEVs performed remarkably with a very large predictive effect with HRs of 2.19 and 2.67 in the discovery and validation cohort, respectively. Comparatively, the effect size outperformed the most potent biomarkers in CAD such as troponin (HR of 1.47 in 10-year follow-up), coronary artery calcium (HR of 2.35 in 10-year follow-up), B-type natriuretic peptide (HR of 1.24 in 3-year follow-up), and high-sensitivity C-reactive protein (HR of 1.47 in 3-year follow-up).^35^^,^^36^ Moreover, PAI-1^+^ PEV levels added to clinical risk factors associated with poor prognosis (including age, type 2 diabetes, and acute coronary syndrome) improved the MACE prediction model and remained an independent predictor of 1-year MACE. Our study shows the potential of PAI-1^+^ PEVs as a prognostic biomarker of MACE in an all-comer population undergoing coronary revascularization, but the findings require external validation in another setting prior to broader clinical application.

Our data set now stands as the largest evaluation to date of EVs as a biomarker. Comparatively, the largest EV biomarker study to date before the current study was in 200 patients with stable CAD reporting the prognostic value of CD31^+^/Annexin V^+^ EVs for MACE in a 6-year follow-up with an HR of 4.0.^37^ Previous biomarker studies have reported elevated levels of EVs with acute coronary syndrome and end-stage renal disease.^14^^,^^35^^,^^38^^,^^39^ The totality of the data suggest that EVs may serve an unmet clinical need providing robust risk stratification, with PAI-1^+^ PEVs exhibiting the most impactful risk stratification to date. Of note, despite the large effect size, it remains technically challenging to isolate EVs for clinical use.

### Study limitations

First, being a highly predictive biomarker of adverse events in and of itself does not confer causal inferences, and we relied on in vitro models to assess the effect of EVs on thrombosis and VSMC biology, respectively. Second, we are not able to isolate PAI-1^+^ PEV fraction from PEVs without inhibiting PAI-1. Furthermore, all bench-top experiments were performed by using EVs isolated from healthy volunteers. Accordingly, we were not able to directly observe the effect of the complex on VSMC phenotypic switching. Third, despite the strong performance of PAI-1^+^ PEV, it remains technically difficult to isolate these EVs and requires several specialized equipment and technician training. Moreover, it remains challenging to standardize assays measuring EVs for the development and establishment of routine clinical tests.^40^ Fourth, measurement of plasma PAI-1 levels in isolation without PAI-1^+^ PEV is not predictive of MACE, as shown by a prior meta-analysis performed by our group.^8^ Finally, the effect of PAI-1^+^ PEVs on VSMC phenotypic switching is not testable in an in vivo study. To date, animal models do not exist in which we can manipulate EV production or exogenously administer EVs without initiating the host inflammatory response. Therapeutic development of these observations will likely rely on PAI-1 inhibition, a strategy previously shown to be efficacious in mouse models of arterial injury.^12^^,^^41^

## Conclusions

We report the existence of a PAI-1^+^ PEV complex in humans that functions as a modulator of proinflammatory changes of neointimal formation in smooth muscle cells. Moreover, PAI-1^+^ PEVs can be used as a powerful biomarker to identify high-risk patients with CAD. Finally, given the attenuation of VSMC phenotypic switching through the inhibition of the PAI-1:LRP1 response, our study suggests PAI-1 as a potential biomarker to identify and a therapeutic target to ameliorate neointimal formation of high-risk patients.

Perspectives **COMPETENCY IN MEDICAL KNOWLEDGE:** Revascularization through PCI is associated with an 8% to 12% annualized event rate, with complications such as in-stent restenosis and stent thrombosis. In this study, we identified a novel biomarker in the form of PAI-1^+^ PEV as a biomarker in patients for 1-year MACE. Moreover, we found that PAI-1^+^ PEVs promote the proinflammatory VMSC phenotype and that this interaction is largely driven by the PAI-1:LRP1 interaction. This study shows the potential of PAI-1^+^ PEVs as a therapeutic target and biomarker to explore in larger clinical applications.

**TRANSLATIONAL OUTLOOK 1:** PAI-1^+^ PEVs are a promising biomarker for 1-year MACE after coronary angiography with better performance than currently used clinical biomarkers. Future external validation studies should be performed before broader clinical application.

**TRANSLATIONAL OUTLOOK 2:** Our data show that PAI-1^+^ PEVs modulate the proinflammatory VSMC phenotype mediated by the PAI-1:LRP1 interaction. Inhibition of the interaction through TM5275 disrupted the inflammatory changes and serves to be a novel therapeutic target to reduce neointimal formation.

## Funding Support and Author Disclosures

This project was supported by the Innovation Fund of the Alternative Funding Plan for the Academic Health Sciences Centres of Ontario and the Canadian Foundation of Innovation. Dr Jung was funded by the Vanier Canadian Institutes of Health Research Canada Graduate Scholarship for his graduate studies. Dr Duchez was supported by the Cardiac Endowment Fund at the University of Ottawa Heart Institute. The authors have reported that they have no relationships relevant to the contents of this paper to disclose.
