# Supplementary material for: Plasminogen Activator Inhibitor-1–Positive Platelet-Derived Extracellular Vesicles Predicts MACE and the Proinflammatory SMC Phenotype
Source: JACC Basic Transl Sci. 2022 Sep 21;7(10):985–97. doi: 10.1016/j.jacbts.2022.05.002 (PMC9626902; doi:10.1016/j.jacbts.2022.05.002)
Supplement: Supplemental Data [file mmc1.pdf]

Supplemental Material

Plasminogen activator inhibitor-1 platelet extracellular vesicles predicts MACE and the pro-inflammatory SMC phenotype

Richard G. Jung MD PhD,[1,2,3] Anne-Claire Duchez PhD,[4] Trevor Simard MD,[1,2,3,5] Shan Dhaliwal BSc,[1,2,4,6] Taylor Gillmore MSc,[7] Pietro Di Santo MD,[1,2,4,6] Alisha Labinaz BSc,[1,2] F. Daniel Ramirez MD MSc,[1,2,5] Adil Rasheed PhD,[4] Sabrina Robichaud BSc,[4] Mireille Ouimet PhD,[4] Spencer Short BSc BASc,[7] Cole Clifford MD,[7] Fengxia Xiao PhD,[8] Marie Lordkipanidzé BPharm PhD, [9,10] Dylan Burger PhD,[8] Suresh Gadde PhD,[4] Katey J. Rayner PhD,[4] and Benjamin Hibbert MD PhD[1,2,3,5]

1. CAPITAL Research Group, University of Ottawa Heart Institute, Ottawa, Ontario, Canada
2. Vascular Biology and Experimental Medicine Laboratory, University of Ottawa Heart Institute, Ottawa, Ontario, Canada
3. Department of Cellular and Molecular Medicine, Faculty of Medicine, University of Ottawa, Ottawa, Ontario, Canada
4. Department of Biochemistry, Microbiology, and Immunology, Faculty of Medicine, University of Ottawa, Ottawa, Ontario, Canada
5. Division of Cardiology, University of Ottawa Heart Institute, Ottawa, Ontario, Canada
6. School of Epidemiology and Public Health, University of Ottawa, Ottawa, Ontario, Canada
7. Faculty of Medicine, University of Ottawa, Ottawa, Ontario, Canada
8. Kidney Research Centre, Department of Cellular and Molecular Medicine, Faculty of Medicine, University of Ottawa, Ontario, Canada
9. Faculté de Pharmacie, Université de Montréal, Montréal, Québec, Canada
10. Research Center, Montreal Heart Institute, Montréal, Québec, Canada

Supplemental Methods

Participant selection

Study population and sample collection

The University of Ottawa Heart Institute is a regional tertiary center serving over 1.2 million people in the capital region of Canada and all coronary catheterization procedures are registered in the Cardiovascular And Percutaneous clInical TriALs (CAPITAL) Revascularization Registry.(1) The CAPITAL Revascularization Registry is a prospective registry capturing over 1200 clinical datapoints and has been previously described in detail elsewhere.(2) Concurrently, between October 2016 to August 2018, blood samples were collected on consecutively eligible patients referred for coronary revascularization immediately after the completion of the procedure and processed as previously described by our group.(2)

Clinical outcomes

Outpatient clinic or chart review was conducted at one-year following baseline blood draw to evaluate the endpoints of MACE and unplanned revascularization. The primary outcome was MACE, defined as a composite of all-cause mortality, myocardial infarction, unplanned revascularization, and cerebrovascular accident. Secondary outcomes were individual components of MACE. Clinical follow-up was conducted in-person and recorded in the CAPITAL Revascularization Registry. (2) Clinicians who performed the clinical follow-up were blinded to PAI-1^+ PEV levels.

Identification of discovery and validation cohort

Participants in both the discovery and validation cohort in the study include ≥18 years of age referred to the University of Ottawa Heart Institute for coronary angiography and/or revascularization with conventional coronary angiography or CT angiography confirmed diagnosis of coronary artery disease. Exclusion criteria include unwillingness or inability to provide informed consent, anemia preventing blood draws, and when the blood sample was exhausted.

Between August 2015 to October 2018, 1851 patients who were referred for coronary angiography had their blood collected. Patients were excluded with incomplete clinical follow-up and missing data (n=726) and samples used in other studied (n=669). 456 patients had their PAI-1^{+} PEV levels measured. The entire cohort was then equally split into a discovery or training data (n=228) and validation group (n=228) following simple random sampling of MACE events (n=76; 38 in each group) and non-MACE events (n=380; 190 in each group) using SAS v9.4.

Plasma PAI-1 assessment

Human plasma spun at 3,200 or 20,000 x g at 4°C were measured in a 1:5 dilution using human PAI-1 enzyme-linked immunosorbent assay was purchased from Abcam (ab184863, Abcam, Cambridge, United Kingdom) as per manufacturer's recommendations by a researcher blinded to patient and procedural characteristics. PAI-1 activity (IU/mL) was evaluated as per manufacturer's recommendations using a chromogenic assay kit (ab108894, Abcam).

Electron microscopy

PEVs ($1.0x10^7$ EVs/mL) were incubated for 4 hours in PBS containing 0.05% bovine serum albumin and anti-PAI-1 monoclonal antibody (1:200). Following primary antibody incubation, the sample was washed with PBS and incubated for 1 hour in goat anti-mouse IgG gold-linked polyclonal antibody (1:50, ab27421, Abcam). The sample was washed in PBS and loaded onto carbon-coated copper grids (300 mesh) for transmission electron microscopy (Tecnai^{TM} G^2 Spirit TWIN, FEI Company, Hillsboro, USA).

Human coronary artery smooth muscle cell assessment

Cell culture

Human coronary artery smooth muscle cells (C0175C, ThermoScientific) were grown in Medium 231 (ThermoScientific) supplemented with penicillin-streptomycin and smooth muscle cell growth supplement (S00725), containing 4.9% v/v fetal bovine serum, 2 ng/mL human basic fibroblast growth factor, 0.5 ng/mL human epidermal growth factor, 5 ng/mL heparin, 0.01 ug/mL recombinant human insulin-like growth factor-I, and 0.2 ug/mL BSA at 37°C with 5.0% CO_2. $1.0x10^8$ washed platelets/mL isolated from healthy volunteers were stimulated overnight with 1.0 μg/mL collagen (prepared from addition of 1 mg/mL collagen (P/N 385, Chronolog)) at room temperature in order to generate PEVs and quantified via flow cytometry. VSMCs were differentiated in differentiation supplement (S0085, ThermoScientific) for 72 hours in 6-wells and incubated with extracellular vesicles for 24 hours prior to extraction.

Washed platelet preparation

Whole blood was spun at 200 x g for 20 minutes with no brakes and the top layer was carefully transferred into a falcon tube, PGE_1 was added to a final concentration of 1μM and was spun at 1,000 x g for 10 minutes with no brakes. Platelets were washed in washing buffer (103 mM NaCl, 5 mM KCl, 1 mM $MgCl_2$, 5 mM glucose, 36 mM citric acid, 3.5 mg/mL BSA, and pH 6.5) and spun at 1,000 x g for 10 minutes. Finally, platelets were resuspended in Tyrode's buffer (5 mM HEPES, 137 mM NaCl, 2 mM KCl, 1 mM $MgCl_2$, 12 mM $NaHCO_3$, 0.3 mM NaH_2PO_4, 3.5 mg/mL BSA, and pH 7.4).

Real-time gene expression analysis

VSMCs were plated onto 6-well plates in Medium 231 and were subsequently treated with increasing concentrations of EVs (1.5-6.0x10^4 EVs/μL) and total RNA was extracted using TRIzol LS (10296010, ThermoScientific) at the indicated timepoints according to manufacturer's recommendations. Total RNA (750 ng) was used for reverse transcription using SuperScript™ IV VILO Master Mix (11766050, ThermoScientific) according the manufacturers' guidelines. Following reverse transcription, cDNA diluted 1:10 was used for real-time PCR analysis using SYBR Select Master Mix for CFX (4472942, ThermoScientific) according to manufacturer's guideline. Briefly, the real-time was run for 40 cycles at 95 °C denaturing for 15 seconds, 55°C annealing for 15 seconds, and 72°C extension for 1 minute using the Eppendorf RealPlex2 machine and quantified by the Pfaffl method.(3) Primers for *KLF4, KLF5*, smooth muscle cell actin (*ACTA2*), and *GAPDH* were previously described.(4)

Cell proliferation and apoptosis

VSMC proliferation was measured using the CellTrace Violet kit (C34557, ThermoScientific). VSMCs were stained in the presence of 5 μM CellTrace Violet were seeded onto 96-well plates (5.0x10^3 VSMCs/mL) for 24 hours. They were then washed with PBS and incubated with increasing concentrations of PEVs (1.5-10.0x10^4 EVs/μL) and/or tiplaxtinin (SigmaAldrich) for 24 and 48 hours at 37°C and 5.0% CO_2. Proliferation and apoptosis (Annexin V-FITC kit, 130-092-052, Miltenyi Biotec) was quantified according to manufacturer's recommendations using the MACSQuant Analyzer 10.

Scratch assay

VSMCs were plated onto 96-well plates (2x10^4 cells/mL) in Medium 231 and incubated at 37°C and 5.0% CO_2 until confluence was achieved. VSMCs were then labelled with CellMask Orange (C10045, ThermoScientific) and washed with PBS. Cells were scratched using a p200 tip in a vertical direction, washed with PBS, and treatments suspended in Medium 231 were added to the 96-well plates and imaged every 2 hours using the Cytation 5 (BioTek, Winooski, Vermont, USA). Wound closure percentage was quantified on the Cytation Gen5 software using manufacturer recommendations.

Extracellular vesicle analysis

Flow cytometry

For isolation of EVs, plasma was first spun at 20,000 x g for 20 minutes. Then the sample was resuspended in Annexin V buffer and incubated with Annexin V-PE (130-118-363, Miltenyi Biotec), spun at 20,000 g for 20 minutes, and incubated with appropriate antibodies (1:20 CD41a-VioBlue (130-105-558) and 1:7.5 PAI-1-FITC (11501-05041, AssayPro) or IgG-FITC for 1 hour.

Samples were spun at 20,000 g for 20 minutes and resuspended in phosphate-buffered saline (**PBS**), with 1:300,000 1.00 μm Fluoresbrite YG Microspheres (17154-10, Polysciences Inc). Data on 10,000 YG microsphere events were acquired using the MACSQuant 10 flow cytometer (Miltenyi Biotec). PAI-1$^+$ PEV (defined as Annexin V$^+$/CD41$^+$/PAI-1$^+$) quantification was conducted using FlowJo v10 (Ashland, Oregon, USA).

Thrombus formation using the Total Thrombus Analysis System

Thrombus formation was analyzed using a validated automatic microchip-based flow chamber system Total Thrombus Analysis System (**T-TAS**, Zacros, Fujimori Kogyo Company, Japan), using the AR chip.(5-8) Whole blood was incubated with increasing concentrations of EVs isolated from volunteers for 5 minutes prior to T-TAS analysis. For comparative analysis between ISR and control EV, plasma from ISR and control patients were spun at 20,000g for 90 minutes and a final concentration of 3.5x10^4 EVs/uL was added to volunteer whole blood prior to analysis on T-TAS. Differences in thrombus formation was quantified at four points: 1) time to thrombus onset measured at 10 kPa (**T_{10}**), 2) time to occlusive thrombus measured at 80 kPa (**T_{80}**), 3) the rate of thrombus formation measured as a difference of T_{80} and T_{10}, and 4) total thrombogenicity quantified as area under the pressure vs. time curve.

Human coronary artery smooth muscle cell assessment

Platelet extracellular vesicle interaction with human coronary VSMCs

Platelets (1.0x10^8/mL) were labelled with 10μM CellTracker Green CMFDA Dye (C2925, ThermoScientific) in Tyrode's Buffer for 20 minutes and spun at 1000 x g for 10 minutes without brakes. CMFDA$^+$ platelets were resuspended in Tyrode's Buffer and stimulated with 1.0 μg/mL collagen overnight to generated CMFDA$^+$ platelet EVs and quantified by flow cytometry.

Subsequently, $1x10^8$ platelet EVs/mL were incubated with tiplaxtinin or TM5275 for 30 minutes at room temperature. Subsequently, they were co-incubated with human coronary VSMCs for 40 minute at room temperature as previously described by another group.(9) Free platelet EVs were removed following centrifugation at 1400 rpm and washed with PBS. Control incubation was done by co-incubating PEVs with VSMCs in both vehicle control or in the presence of EDTA. $CMFDA^+$ EV interaction with VSMC was quantified by median VSMC FITC intensity by flow cytometry.

Internalization of platelet extracellular vesicles into smooth muscle cells

Washed platelets ($1.0x10^8$ /mL) extracted from healthy volunteers was stained with CFSE for 20 minutes in Tyrode's buffer and spun at 3,200 x g for 20 minutes. Labelled platelets were subsequently stimulated with 1.0 μg/mL collagen overnight and platelets EVs were quantified using Annexin V and CD41. CellMask Orange labelled VSMCs plated on glass slips were co-incubated with CellTracker $Green^+$ EVs for 8, 12, and 24 hours and fixed with 4% paraformaldehyde and labelled with Hoescht staining dye. Three-dimensional images were acquired using the Zeiss Elyra LSM880 and analyzed using the Zen lite 3.1 and ImageJ.

Supplemental Table 1. List of primers used for real-time analysis

Target Gene	Sequence
KLF4 FWD	CCCACATGAAGCGACTTCCC
KLF4 REV	CAG GTC CAG GAG ATC GTT GAA
KLF5 FWD	TCA GTC GTA GAC CAG TTC TTC A
KLF5 REV	CTG GGA TTT GTA GAG GCC AGT
GAPDH FWD	CAT GAG AAG TAT GAC AAC AGC CT
GAPDH REV	AGT CCT TCC ACG ATA CCA AAG T
ACTA2 FWD	GTG TTG CCC CTG AAG AGC AT
ACTA2 REV	GCT GGG ACA TTG AAA GTC TCA
MMP-2 FWD	CAG GGA ATG AGT ACT GGG TCT ATT
MMP-2 REV	ACT CCA GTT AAA GGC AGC ATC TAC
Osteocalcin FWD	CGC TAC CTG TAT CAA TGG CTG G
Osteocalcin REV	CTC CTG AAA GCC GAT GTG GTC A
BMP-2 FWD	TGT ATC GCA GGC ACT CAG GTC A
BMP-2 REV	CCA CTC GTT TCT GGT AGT TCT TC
CCL2 FWD	AGA ATC ACC AGC AGC AAG TGT CC
CCL2 REV	TCC TGA ACC CAC TTC TGC TTG G

Supplemental Table 2. List of materials used for the study

Material	Company	Catalogue Number
Prostaglandin E1	Sigma-Aldrich	P5515
PAI-1 ELISA	Abcam	ab184863
PAI-1 activity kit	Abcam	ab108894
PAI-1 monoclonal antibody (1D5)	Invitrogen	MA517171
Goat anti-mouse IgG gold-linked polyclonal antibody	Abcam	ab27421
Human coronary artery smooth muscle cells	ThermoScientific	C0175C
Medium 231	ThermoScientific	M231500
Smooth muscle cell growth supplement	ThermoScientific	S00725
Smooth muscle cell differentiation supplement	ThermoScientific	S0085
Penicillin-Streptomycin (10000 U/mL)	Gibco	15140122
Collagen	Chrono-Log	P/N 385
TRIzol LS	ThermoScientific	10296010
SuperScript IV VILO Master Mix	ThermoScientific	11766050
CellTrace Violet	ThermoScientific	C34557
Tiplaxtinin	Sigma-Aldrich	PZ0295
TM5275	Sigma-Aldrich	SML1398
Annexin V-FITC	Miltenyi Biotec	130-118-363
SYBR Select Master Mix for CFX	ThermoScientific	4472942
CellMask Orange	ThermoScientific	C10045
CD41a-VioBlue	Miltenyi Biotec	130-105-558
PAI-1 FITC	AssayPro	11501-05041
1.0 um Fluoresbrite YG Microsphere	Polysciences Inc.	17154-10
AR Chip for T-TAS	Fujimori Kogyo	19001
CaCTI reagent for T-TAS	Fujimori Kogyo	19004
PL Chip for T-TAS	Fujimori Kogyo	18002
CellTracker Green CMFDA Dye	ThermoScientific	C2925

Supplemental Table 3. Differences in baseline characteristics by major adverse cardiac events

	No MACE (n=380)	MACE (n=76)	P-value
PAI-1⁺ PEV (/uL) – median (Q1-Q3)	8345 [3777-33500]	13787 [6712-26942]	0.047
Age - mean (SD)	65.5 (11.1)	72.5 (10.9)	<0.0001
Sex (female) - no. (%)	100 (26.3)	24 (31.6)	0.35
Hypertension - no. (%)	250 (65.8)	52 (68.4)	0.66
Dyslipidemia - no. (%)	221 (58.2)	56 (73.7)	0.011
Diabetes - no. (%)	112 (29.9)	35 (46.1)	0.006
Smoking - no. (%)			0.37
Never	226 (59.5)	39 (51.3)	
Remote (quit >1 month ago)	89 (23.4)	23 (30.3)	
Active	65 (17.1)	14 (18.4)	
Family history of CAD - no. (%)	55 (14.5)	10 (13.2)	0.76
Atrial fibrillation - no. (%)	37 (9.7)	11 (14.5)	0.22
Indications for angiography - no. (%)			
Acute coronary syndrome	165 (43.4)	38 (50.0)	0.29
Staged percutaneous coronary intervention	32 (8.4)	10 (13.2)	0.19
Stable coronary artery disease	144 (37.9)	21 (27.6)	0.089
Shock	1 (0.3)	0 (0.0)	0.65
Previous history - no. (%)			
Percutaneous coronary intervention	96 (25.3)	22 (29.0)	0.50
Myocardial infarction	63 (16.6)	22 (29.0)	0.012
Coronary artery bypass grafting	14 (3.7)	9 (11.8)	0.003
Peripheral artery disease	25 (6.6)	11 (14.5)	0.020
Cerebrovascular accident	19 (5.0)	9 (10.5)	0.062

Bleed	7 (1.8)	1 (1.3)	0.75
Heart failure	19 (5.0)	4 (5.3)	0.92
Medications - no. (%)			
Aspirin	346 (91.1)	69 (90.8)	0.94
P2Y12 inhibitor	343 (90.3)	67 (88.2)	0.58
ACEi/ARB	199 (52.4)	39 (51.3)	0.87
Beta blocker	223 (58.7)	45 (59.2)	0.93
Calcium channel blocker	48 (12.6)	15 (19.7)	0.10
Statin	307 (80.8)	54 (85.5)	0.33
Proton pump inhibitor	56 (14.7)	15 (19.7)	0.27
Number of vessels with obstructive (≥ 50%) CAD - no. (%)			0.49
0	86 (22.6)	13 (17.1)	
1	157 (41.3)	36 (47.4)	
2	137 (36.1)	27 (35.5)	
Revascularized (PCI + CABG) - no. (%)	235 (61.8)	35 (46.1)	0.011

BMI – body mass index; CAD – coronary artery disease; ACEi/ARB – angiotensin converting enzyme inhibitor/angiotensin ii receptor blocker.

Supplemental Table 4. Baseline characteristics by discovery and validation cohort

	**Total (n=456)**	**Discovery (n=228)**	**Validation (n=228)**
Age - mean (SD)	66.7 (11.3)	66.9 (11.4)	66.5 (11.3)
Sex (female) - no. (%)	124 (27.2)	60 (26.3)	64 (28.1)
Hypertension - no. (%)	302 (66.2)	157 (68.9)	145 (63.6)
Dyslipidemia - no. (%)	277 (60.8)	148 (64.9)	129 (56.6)
Diabetes - no. (%)	147 (32.2)	73 (32.3)	74 (32.9)
Smoking - no. (%)			
Never	265 (58.1)	134 (58.8)	131 (57.5)
Remote (quit >1 month ago)	112 (24.6)	54 (23.7)	58 (25.4)
Active	79 (17.3)	40 (17.5)	39 (17.1)
Family history of CAD - no. (%)	65 (14.3)	32 (14.0)	33 (14.5)
Atrial fibrillation - no. (%)	48 (10.5)	24 (10.5)	24 (10.5)
Indications for angiography - no. (%)			
Acute coronary syndrome	203 (44.5)	96 (42.1)	107 (46.9)
Staged percutaneous coronary intervention	42 (9.2)	19 (8.3)	23 (10.1)
Stable coronary artery disease	165 (36.2)	87 (38.2)	78 (34.2)
Shock	1 (0.2)	0 (0.0)	1 (0.4)
Previous history - no. (%)			
Percutaneous coronary intervention	118 (25.9)	57 (25.0)	61 (26.8)
Myocardial infarction	85 (18.6)	44 (19.3)	41 (18.0)
Coronary artery bypass grafting	23 (5.0)	10 (4.4)	13 (5.7)
Peripheral artery disease	36 (7.9)	22 (9.7)	14 (6.1)
Cerebrovascular accident	27 (5.9)	15 (6.6)	12 (5.3)
Bleed	8 (1.8)	3 (1.3)	5 (2.2)
Heart failure	23 (5.0)	12 (5.3)	11 (4.8)
Medications - no. (%)			
Aspirin	415 (91.0)	212 (93.0)	203 (89.0)
P2Y12 inhibitor	410 (89.9)	205 (89.9)	205 (89.9)
ACEi/ARB	238 (52.2)	114 (50.0)	124 (54.4)
Beta blocker	268 (58.8)	136 (59.7)	132 (57.9)
Calcium channel blocker	63 (13.8)	30 (13.2)	33 (14.5)
Statin	372 (81.6)	194 (85.1)	178 (78.1)
Proton pump inhibitor	71 (15.6)	41 (18.0)	30 (13.2)
Number of vessels with obstructive (≥ 50%) CAD - no. (%)			
0	99 (21.7)	49 (21.5)	50 (21.9)
1	193 (42.3)	98 (43.0)	95 (41.7)
2	164 (36.0)	81 (35.5)	83 (36.4)
Revascularized (PCI + CABG) - no. (%)	270 (59.2)	136 (59.7)	134 (58.8)

References

1. Jung RG, Simard T, Di Santo P et al. Performance of plasminogen activator inhibitor-1 as a biomarker in patients undergoing coronary angiography: Analytical and biological considerations. Diabetes and Vascular Disease Research 2019:1479164119845123.
2. Jung RG, Simard T, Di Santo P et al. Evaluation of plasminogen activator inhibitor-1 as a biomarker of unplanned revascularization and major adverse cardiac events in coronary angiography and percutaneous coronary intervention. Thrombosis Research 2020.
3. Jung R, Radko S, Pelka P. The Dual Nature of Nek9 in Adenovirus Replication. J Virol 2016;90:1931-43.
4. Zeng Z, Xia L, Fan X et al. Platelet-derived miR-223 promotes a phenotypic switch in arterial injury repair. The Journal of Clinical Investigation 2019;129:1372-1386.
5. Al Ghaithi R, Mori J, Nagy Z et al. Evaluation of the Total Thrombus-Formation System (T-TAS): application to human and mouse blood analysis. Platelets 2019;30:893-900.
6. Ito M, Kaikita K, Sueta D et al. Total Thrombus-Formation Analysis System (T-TAS) Can Predict Periprocedural Bleeding Events in Patients Undergoing Catheter Ablation for Atrial Fibrillation. Journal of the American Heart Association;5:e002744.
7. Sueta D, Kaikita K, Okamoto N et al. A novel quantitative assessment of whole blood thrombogenicity in patients treated with a non-vitamin K oral anticoagulant. Int J Cardiol 2015;197:98-100.
8. Hosokawa K, Ohnishi T, Kondo T et al. A novel automated microchip flow-chamber system to quantitatively evaluate thrombus formation and antithrombotic agents under blood flow conditions. J Thromb Haemost 2011;9:2029-37.
9. Vajen T, Benedikter BJ, Heinzmann ACA et al. Platelet extracellular vesicles induce a pro-inflammatory smooth muscle cell phenotype. Journal of Extracellular Vesicles 2017;6:1322454.

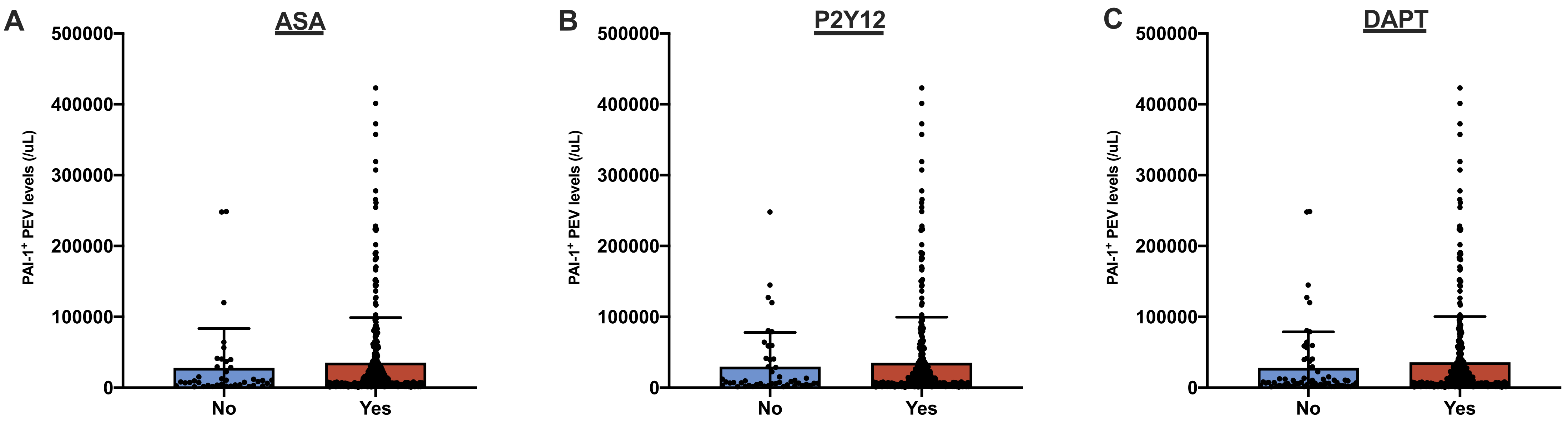

Supplemental Figure 1. PAI-1$^+$ PEV levels stratified by antiplatelet agent. **(A)** Aspirin (ASA) did not affect PAI-1 PAI-1$^+$ PEV levels. **(B)** P2Y12 inhibitor did not affect PAI-1$^+$ PEV levels. **(C)** Dual antiplatelet therapy (DAPT) did not affect PAI-1$^+$ PEV levels. No – refers to patients who are not on the respective antiplatelet therapy. Comparisons were done by Mann-Whitney U-test.

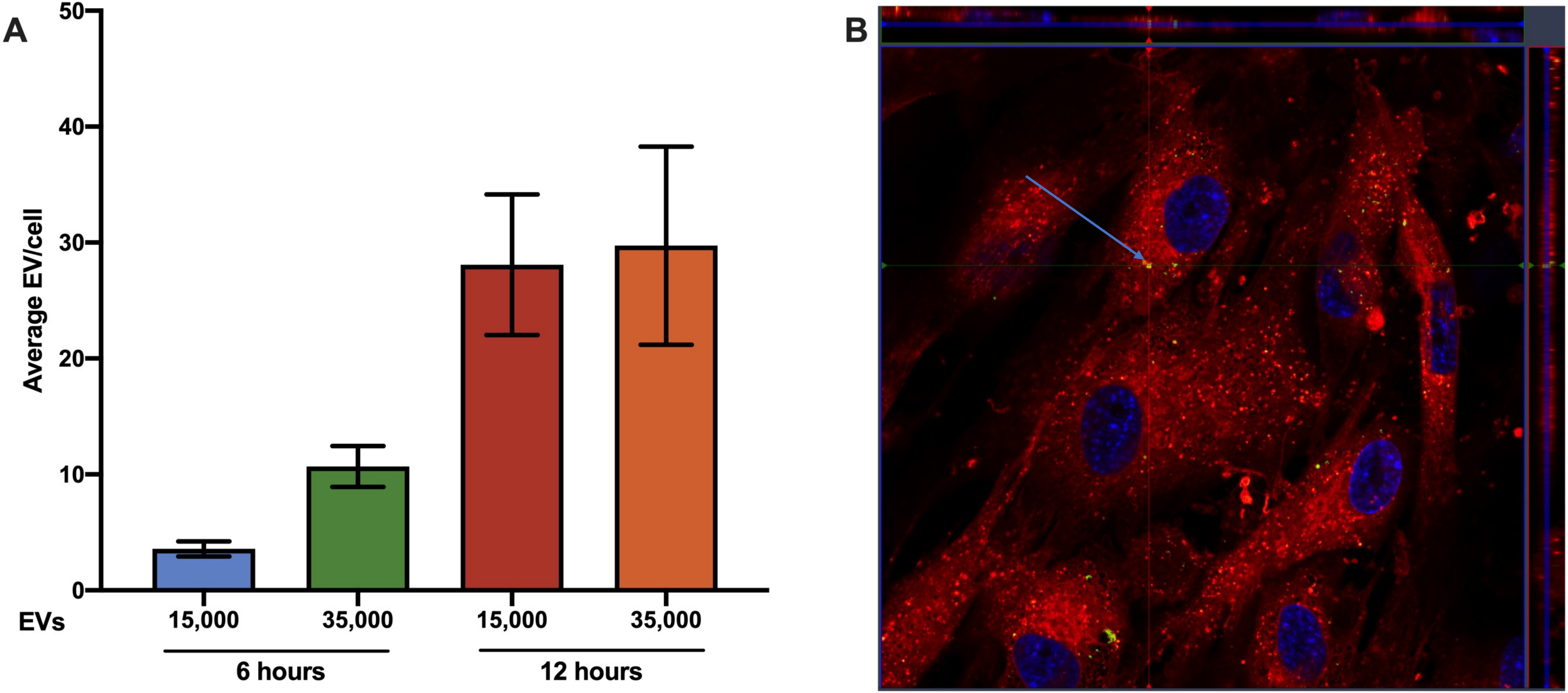

Supplemental Figure 2. Interaction between PEV and VSMCs. **(A)** Incubation with increasing concentrations of CMFDA$^+$ PEV with VSMCs assessed by flow cytometry reveals increased interaction in a dose- and time- dependent fashion. **(B)** Representative immunofluorescence image to evaluate PEV-VSMC interaction. PEV were stained using CellTracker Green (arrow), VSMC was stained in DAPI and CellMask Orange.

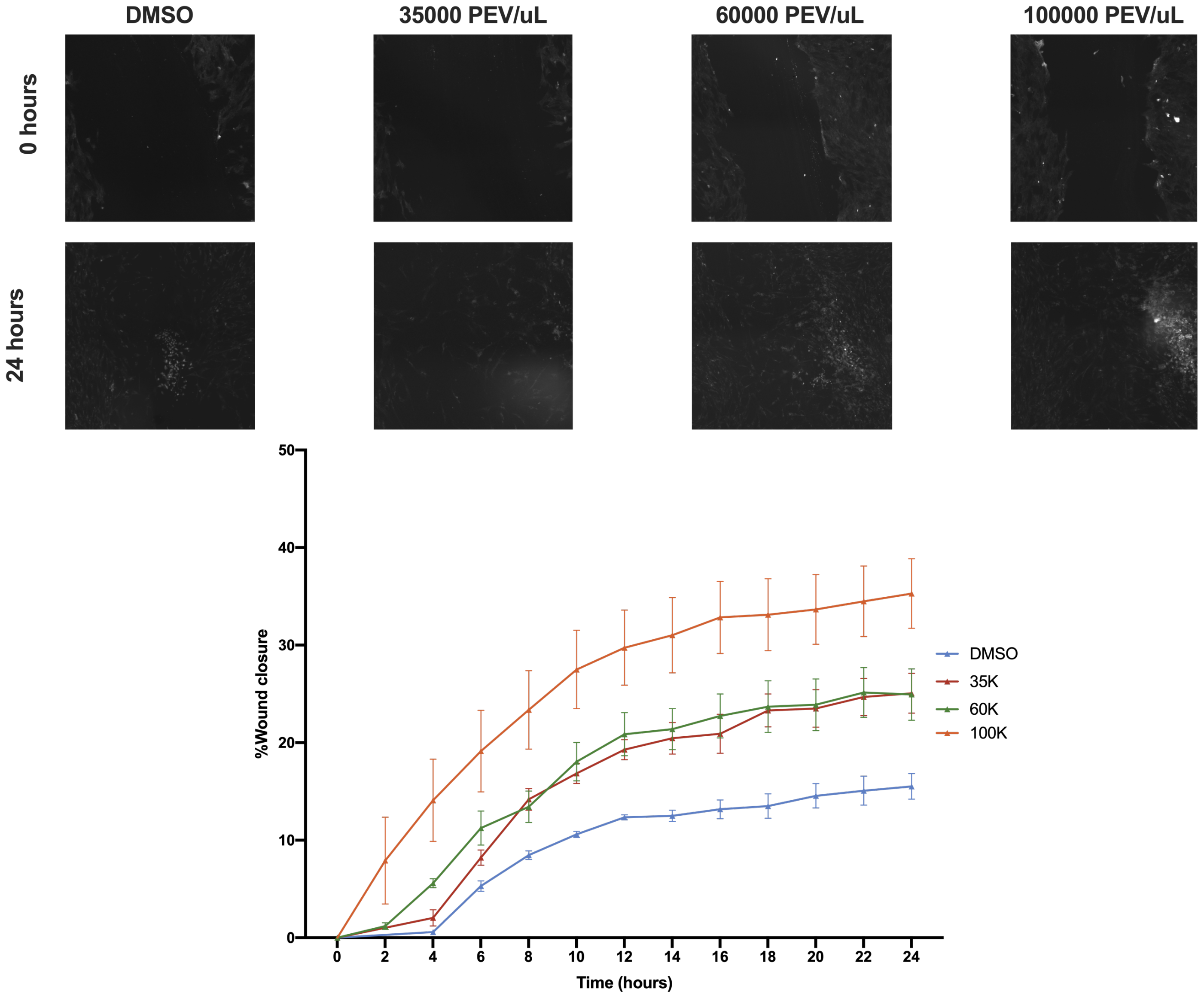

Supplemental Figure 3. Effect of PEV on VSMC migration by scratch assay. Increasing concentrations of PEV enhanced VSMC migration over 24 hours.

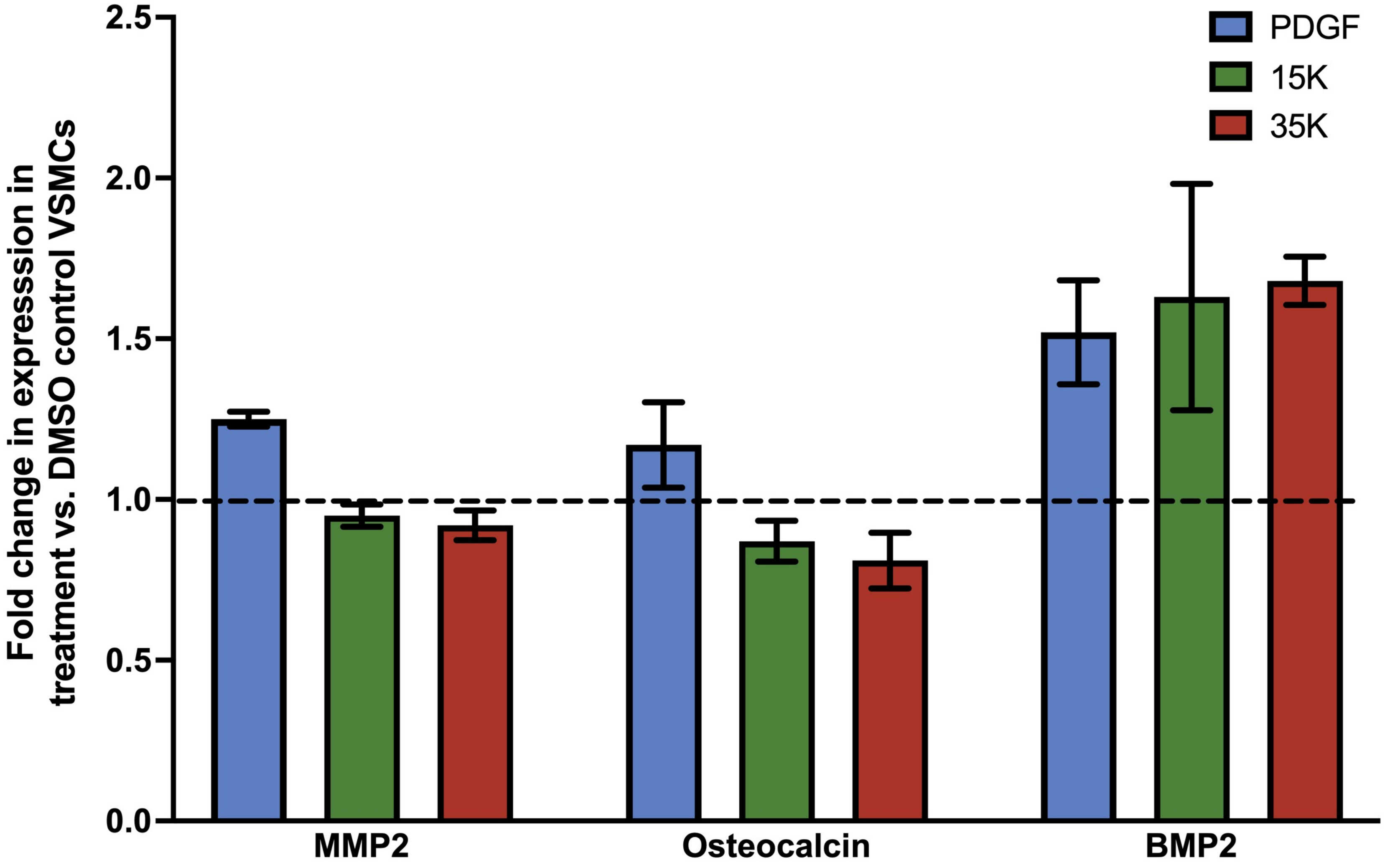

Supplemental Figure 4. Effect of PEV on osteogenic transcription factors. VSMC co-cultured with PEVs exhibited changes in osteogenic marker BMP-2, but not MMP2 or osteocalcin. 15K and 35K represent $1.5x10^4$ PEV/uL and $3.5x10^4$ PEV/uL.

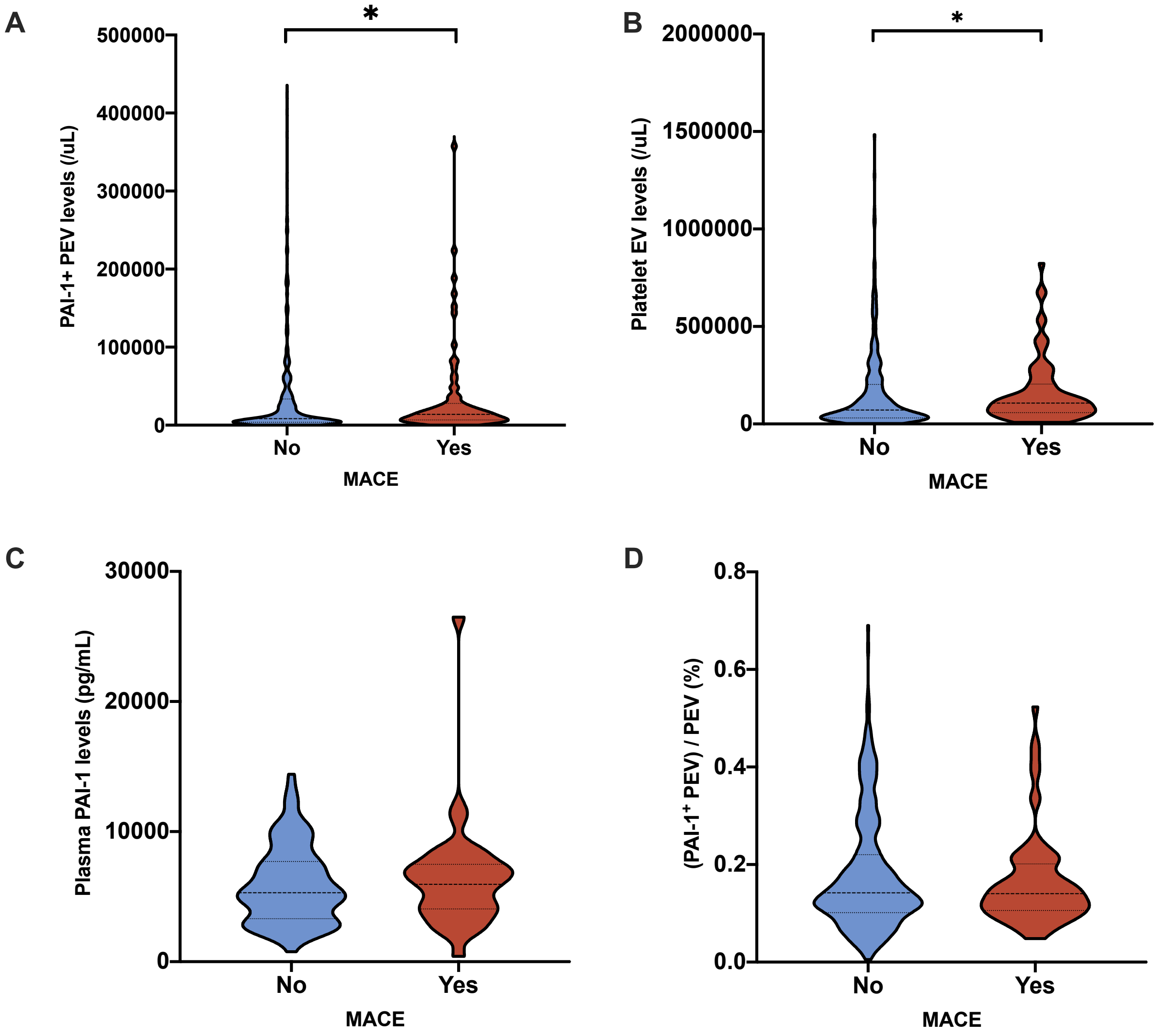

Supplemental Figure 5. Difference in PEV levels (/uL) stratified by MACE. **(A)** Median (Q1-Q3) PAI-1^+ PEV levels were significantly elevated in MACE (13786.6 [6711.7-26942.1] PAI-1^+ PEV/uL vs. 8344.9 [3777.2-33499.7] PAI-1^+ PEV/uL, n=76 and 380, respectively, p=0.047). **(B)** Median (Q1-Q3) PEV levels were elevated in MACE (107463.0 [58416.1-202987.8] PEV/uL vs. 71675.6 [31184.5-201313.3] PEV/uL, n=76 and 380, respectively, p=0.04). (**C**) No differences in plasma PAI-1 levels were observed when stratified by MACE. **(D)** No differences in PAI-1^+ PEV fraction was observed when stratified by MACE. Data are presented as violin graphs and compared by Mann-Whitney U-test. *$p<0.05$.

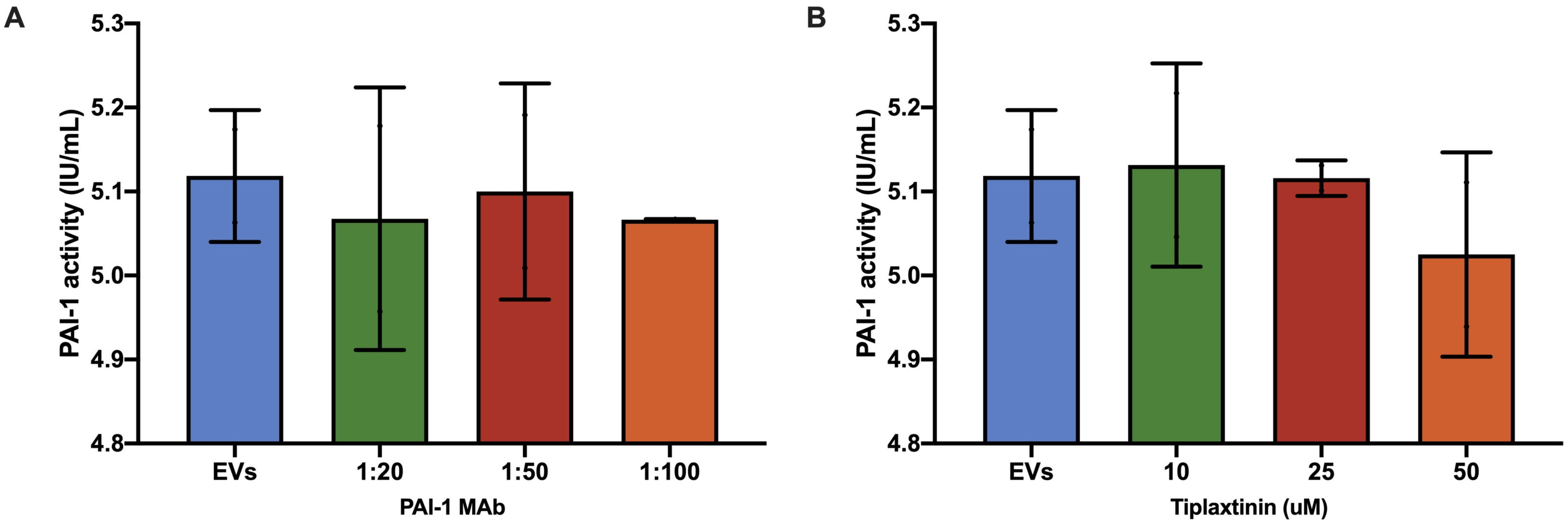

Supplemental Figure 6. PAI-1 activity of PAI-1^{+} PEV complex in the presence of PAI-1 inhibition. **(A)** The addition of PAI-1 monoclonal antibody (MAb) had no effect on activity of PAI-1^{+} PEV complex (IU/mL). **(B)** Increasing concentrations of tiplaxtinin had no effect on PAI-1^{+} PEV complex activity.
